# Preparation of Ruthenium Dithiolene Complex/Polysiloxane Films and Their Responses to CO Gas

**DOI:** 10.3390/molecules23040845

**Published:** 2018-04-07

**Authors:** Satoru Tsukada, Takuya Sagawa, Kazuki Yamamoto, Takahiro Gunji

**Affiliations:** 1 Advanced Materials Laboratory, Advanced Automotive Research Collaborative Laboratory, Graduate School of Engineering, Hiroshima University, 1-4-1 Kagamiyama, Higashi-Hiroshima, Hiroshima 739-8527, Japan; 2 Department of Pure and Applied Chemistry, Faculty of Science and Technology, Tokyo University of Science, 2641 Yamazaki, Noda, Chiba 278-8510, Japan; 7215705@ed.tus.ac.jp (T.S.); kyamamoto@rs.tus.ac.jp (K.Y.); gunji@rs.noda.tus.ac.jp (T.G.)

**Keywords:** ruthenium complex, metalladithiolene complex, carbon monoxide, composite film, sol-gel reaction, tetraethoxysilane

## Abstract

To develop advanced materials using metal complexes, it is better to prepare metal complexes contained in composite or hybrid films. To achieve this purpose, we synthesized ruthenium complexes with dihalogen-substituted benzendithiolate ligands, [(η^6^-C_6_Me_6_)Ru(S_2_C_6_H_2_X_2_)] (X = F, 3,6-Cl, Br, 4,5-Cl), **1b**–**1e**. We also investigated preparation of **1c** or **1e** containing polysiloxane composite films and their reactivity to CO gas. All ruthenium complexes **1b**–**1e** reacted with CO gas, and carbonyl ligand adducts **2b**–**2e** were generated. Ruthenium complexes **1b**–**1e** show two strong absorption peaks around 550 and 420 nm. After exposure to CO gas, these absorption peaks were immediately decreased without a peak shift. A similar trend was observed in **1c** or **1e** containing polysiloxane composite films. These results indicate that **1c** and **1e** were easily converted into **2c** and **2e**, both in the solution and the polysiloxane film during CO gas exposure.

## 1. Introduction

Metal complexes have the potential to lead to the development of innovative materials because of their reactivity, magnetism, and optical and electronic properties. Metal complexes are sometimes hard to handle, due to low stability to light, oxygen, moisture, and/or heat. Additionally, they are typically obtained as a powder or crystal after a reaction. Unfortunately, because of these features, it is difficult to use them directly for the preparation of bulk materials. To develop advanced materials using metal complexes, for example, it is better to prepare metal complexes contained in a polymer [1,2,3,4], composite or hybrid film [5,6,7,8,9], or glass [10,11].

**Scheme 1:**
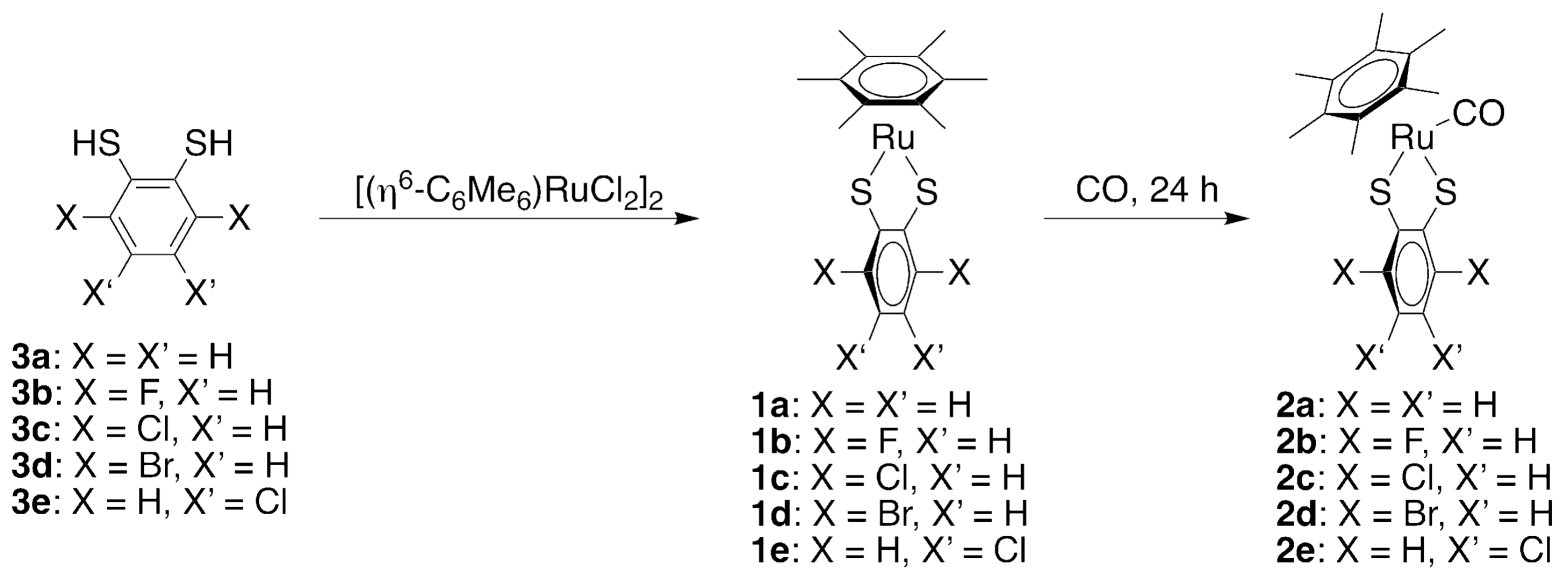
Synthesis of **1a**–**e** and **2b**–**e**.

Carbon monoxide, CO, is one of the more important molecules in C1 chemistry [12,13,14]. CO can be transformed into polymer fibers, plastics, medicines, and bulk chemicals [15]. Therefore, research of adsorption, desorption, purification, and utilization of CO are important. Given this background, the coordination of CO to transition metal complexes has been investigated for over a century. When the CO molecules coordinate to a transition metal center, it can behave as π-acids [16], in addition to σ-donation [17,18]. For example, the study of CO sorption (including the meaning of coordination) of porphyrin complexes [19,20,21,22] and metal-organic frameworks [15,16,17,18] has been carried out. Previously, we reported the addition and dissociation reaction of CO to the ruthenium center of [(*η*^6^-C_6_Me_6_)Ru(S_2_C_6_H_4_)] **1a** in both solution and solid state. This reaction gave CO adduct complex [(*η*^6^-C_6_Me_6_)Ru(S_2_C_6_H_4_)(CO)] **2a**, that have carbonyl ligand on Ru center [23]. We also prepared **1a**/polysiloxane composite films and investigated the reaction of this film with CO gas [23]. This film responded quickly to CO gas with a color change; that is, this film has potential to be a CO gas sensing material. With the Ruthenium complex **1a**, it is possible to introduce substitution groups on benzene rings of benzenedithiolate ligand. Substitution groups may affect the reactivity to CO gas and solubility to a polysiloxane. Therefore, it has been interesting to study the effect of the substitution group on a benzene ring of benzenedithiolate ligand. Here, we report on the synthesis and properties of ruthenium and ruthenium carbonyl complexes with a dihalogen-substituted benzendithiolate ligand (Scheme 1). We also investigated the preparation of ruthenium dithiolene complex/polysiloxane composite films and their reactivity with CO gas.

## 2. Results and Discussion

### 2.1. Synthesis

The starting materials, dihalogen-substituted benzendithiols **3b**–**e**, were prepared using a slightly modified method reported previously [24]. Iron(II) chloride tetrahydrate has a positive impact on the yield compared with iron powder. The yield of these compounds increased more than before. All ruthenium dithiolene complexes **1b**–**e** were successfully synthesized in an almost similar manner to a general method for synthesis of half-sandwich type metalladithiolene complexes [23,24]. The ruthenium carbonyl complex **2a** was obtained quantitatively by the reaction of THF solution of **1a** under a CO gas atmosphere for 6 h [23]. Unfortunately, the yield of **2b**–**e** is low compared with that of **2a** in spite of the long reaction time. This result indicates that disubstituted complexes **2b**–**e** were difficult to react with carbon monoxide because of the low electron density of the ruthenium metal center compared with non-substituted complex **2a**.

### 2.2. Molecular Structures

**Figure 1:**
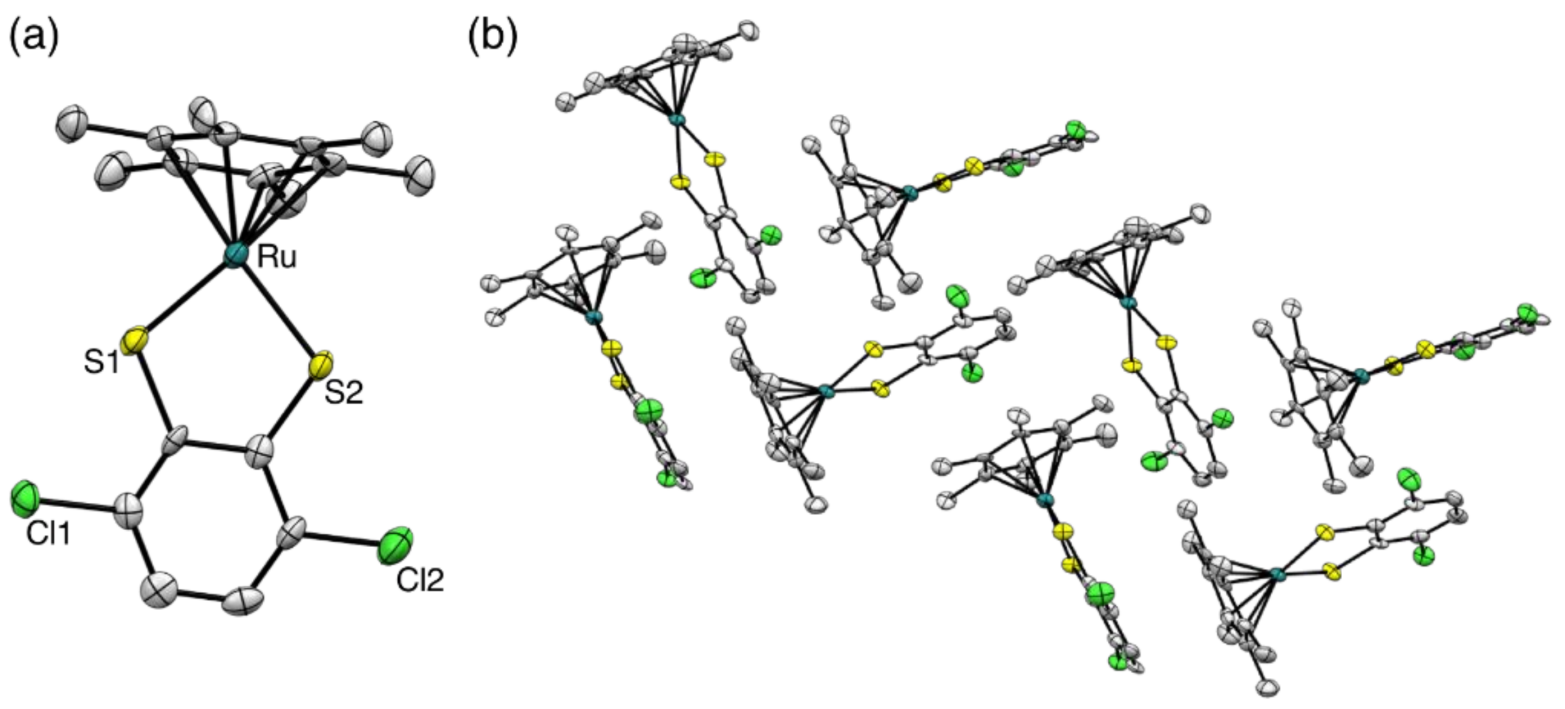
(**a**) ORTEP drawing of **1c** with thermal ellipsoids at the 50% probability level. Hydrogen atoms are omitted for clarity. Color codes: light grey, C; yellow, S; green, Cl; turquoise blue, Ru. (**b**) Packing structure of **1c**.

**Figure 2:**
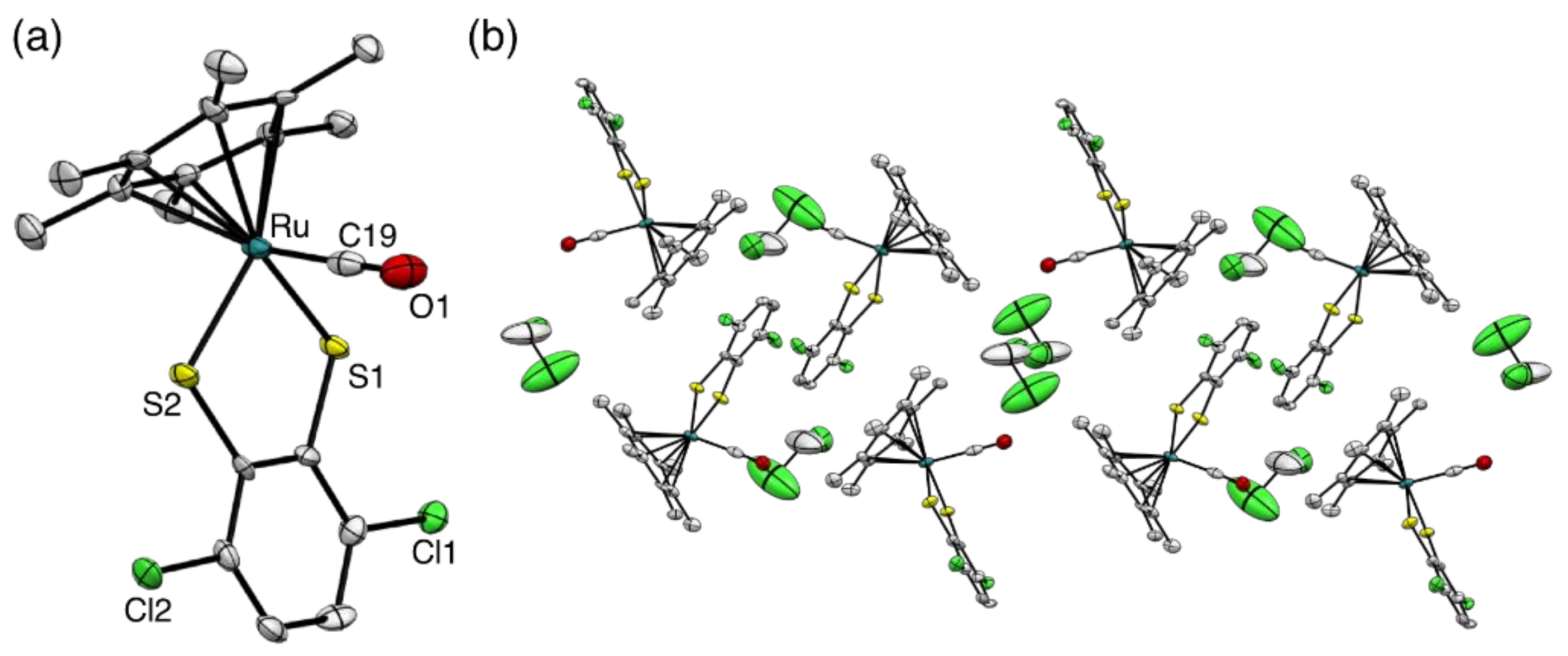
(**a**) ORTEP drawing of **2c** with thermal ellipsoids at the 50% probability level. Hydrogen atoms and dichloromethane molecules are omitted for clarity. Color codes: light grey, C; red, O; yellow, S; green, Cl; turquoise blue, Ru. (**b**) Packing structure of **2c**.

The molecular structures of all complexes were successfully determined by single-crystal X-ray diffraction analysis (Figure 1, Figure 2 and Figures S28–S32). The same packing structures were observed in **1b**–**d**, that is, a staircase pattern arose from the two interactions between a hexamethylbenzene ligand and a dihalogen-substituted benzene ring, organized by right angles at each step (Figure 1b, Figures S28b and S29b). Furthermore, a staircase pattern was arranged in the same direction. This molecular organization is the same as 4,5-dichloro-substituted cobaltadithiolene complex (*η*^5^-C_5_Me_5_)Co(S_2_C_6_H_2_Cl_2_) [24]. On the other hand, the molecular organization of **1e** was the same as **1a**, and slightly different from **1b**–**d**. These complexes show a staircase pattern with a different direction in the next column (Figure S30b). The ruthenium carbonyl complex **2c** was also organized in the same manner as **1e**, with dichloromethane molecules as crystal solvents (Figure 2). The organization of these complexes is comprised of π–π interaction between the C_6_Me_6_ ring and the benzene ring of benzeneditiholate ligand. A unique packing structure was observed in **2d** (Figure S32). A 1,2-dichlorobenzene molecule as a crystal solvent was sandwiched by the two C_6_Me_6_ rings of **2d**, which confronted each other face-to-face. In other words, two molecules and one 1,2-dichlorobenzene molecule form a double-decker structure in single crystals.

The C–O distance of **2a**–**e** (Table 1) did not differ greatly from that of a free CO molecule (1.128 Å) [25]. This result indicated that π back-donation to CO ligand was weak because of low electron density on Ru center by electron withdrawing of halogen atoms. In other words, the Ru–C bond is expected to weaken. The Ru–C–O angle of all carbonyl complexes was close to 180° (Table 1), which indicates that the CO ligand was coordinated to the Ru center in a *η*^1^-coordination fashion. The IR spectrum was also suggested as the coordination mode of CO ligand (Table 1). The carbonyl complexes **2a**–**e** showed only one strong band that was assigned to the stretching vibration of the carbonyl groups associated with the terminal coordination bond (Figure S27). Additionally, **2a** showed the lowest value of CO vibration in the carbonyl complexes. In other words, π back-donation to CO ligand of **2a** seems to be larger compared with the other carbonyl complexes **2b**–**e**. We note that the C–O distance of **2a**–**e** was not according to the result of IR spectra. This result is within the margin of error. The C_6_Me_6_ ligand of **2a**–**e** was inclined from perpendicular coordination mode of **1a**–**e** toward the RuS_2_C_2_ ring. The angle of **2a**–**e** between the C_6_Me_6_ ligand and the RuS_2_C_2_ ring was around 55°; that is, conformation around the central metal was a piano-stool geometry. These structure features of **1a**–**e** with *η*^1^-coordination ligand on ruthenium center resemble those of [(*η*^6^-C_6_Me_6_)Ru(S_2_C_6_H_4_)]_2_(*µ*_2_-NH_2_NH_2_)] [26] and [(*η*^6^-C_6_Me_6_)Ru(CN*t*Bu)(S_2_C_6_H_4_)] [27].

### 2.3. Optical Properties and Thermal Characteristics

Thermogravimetric and differential thermal analysis (TG-DTA) of **2c** indicated that a weight loss (~10%) occurred at 70–104 °C (Figure S35). Then, a large weight loss (~71%) was observed at 283–318 °C. TG-DTA of **1c** show a drastic weight loss at 286–338 °C, which is ascribed to combustion of **1c** (Figure S33). Therefore, both weight losses were ascribed to the release of CO from **2c** to generate **1c**, and then combustion of **1c**, respectively. **2e** and **1e** also show similar behavior (Figures S34 and S36).

**Figure 3:**
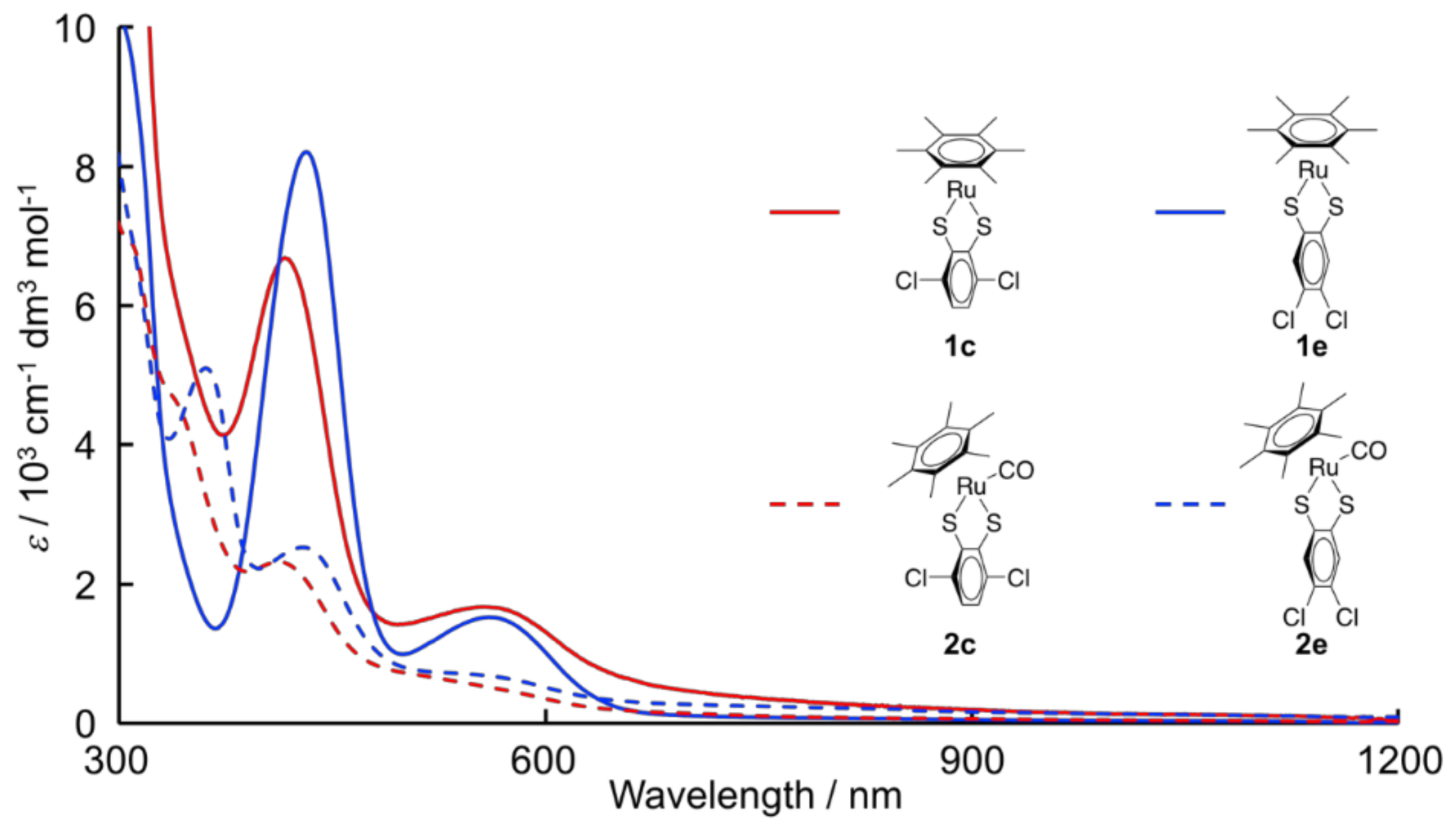
UV-Vis-NIR spectra of **1c**, **1e**, **2c**, and **2e** in THF.

The absorption spectra of **1c** showed two strong peaks at 558 and 417 nm. In carbonyl complex **2c**, each peak was decreasing without a peak shift (Figure 3). The absorption spectra of **1e** and **2e** showed the same trend. These are the same relation between **1a** and **2a** [23]. Therefore, these peaks were attributed in the same manner as **1a** and **2a**. In other words, the strong peaks of **1c** and **1e** around 550 and 420 nm were attributed to the highest occupied molecular orbital (HOMO)—lowest unoccupied molecular orbital (LUMO) and HOMO-1—LUMO transitions, respectively (see results of TD-DFT calculations in supporting information). This HOMO and HOMO-1 were mainly located on the dithiolene ring. The LUMO was based on the d-orbital at the Ru atom. These results indicated that the absorption peaks of **1c** and **1e** were attributed to a ligand-to-metal charge-transfer (LMCT). Alternatively, the HOMO of **2c** and **2e** was almost similar to the LUMO of **1c** and **1e**. This is because the vacant d-orbital of **1c** and **1e** was filled with electrons by the addition of the CO. Therefore, both *ε* of **2c** and **2e** decrease by the structural changes around the Ru atom from the vertical coordination between the C_6_Me_6_ ligand and the dithiolene part to piano-stool geometry. A similar color change (based on a geometry change) was reported as a cobalt dithiolene complex, that is between [(*η*^5^-C_5_H_5_)Co(S_2_C_2_(COOMe)_2_)] and [(*η*^5^-C_5_H_5_)Co(P(OMe)_3_)(S_2_C_2_(COOMe)_2_)] [28].

### 2.4. Preparation of Composite Films and Their Reactivity with CO Gas

**Figure 4:**
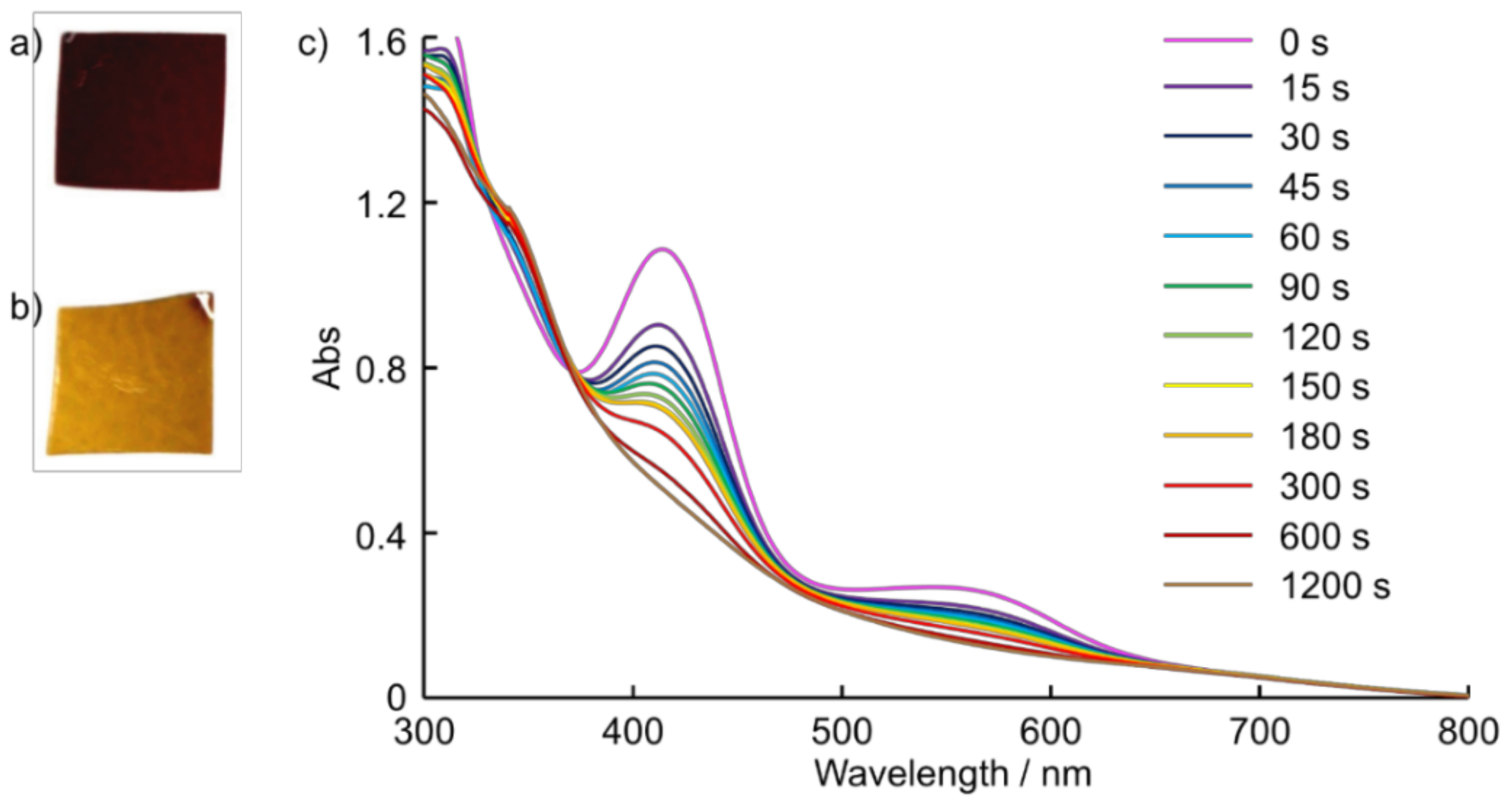
Photographs of (**a**) **1c**/polysiloxane self-standing composite film (thickness: 0.555 mm) and (**b**) **2c**/polysiloxane composite film, which was prepared by exposure of the **1c**/polysiloxane composite to CO gas. (**c**) UV-Vis spectral changes of the **1c**/polysiloxane self-standing composite film during exposure to CO gas.

**Figure 5:**
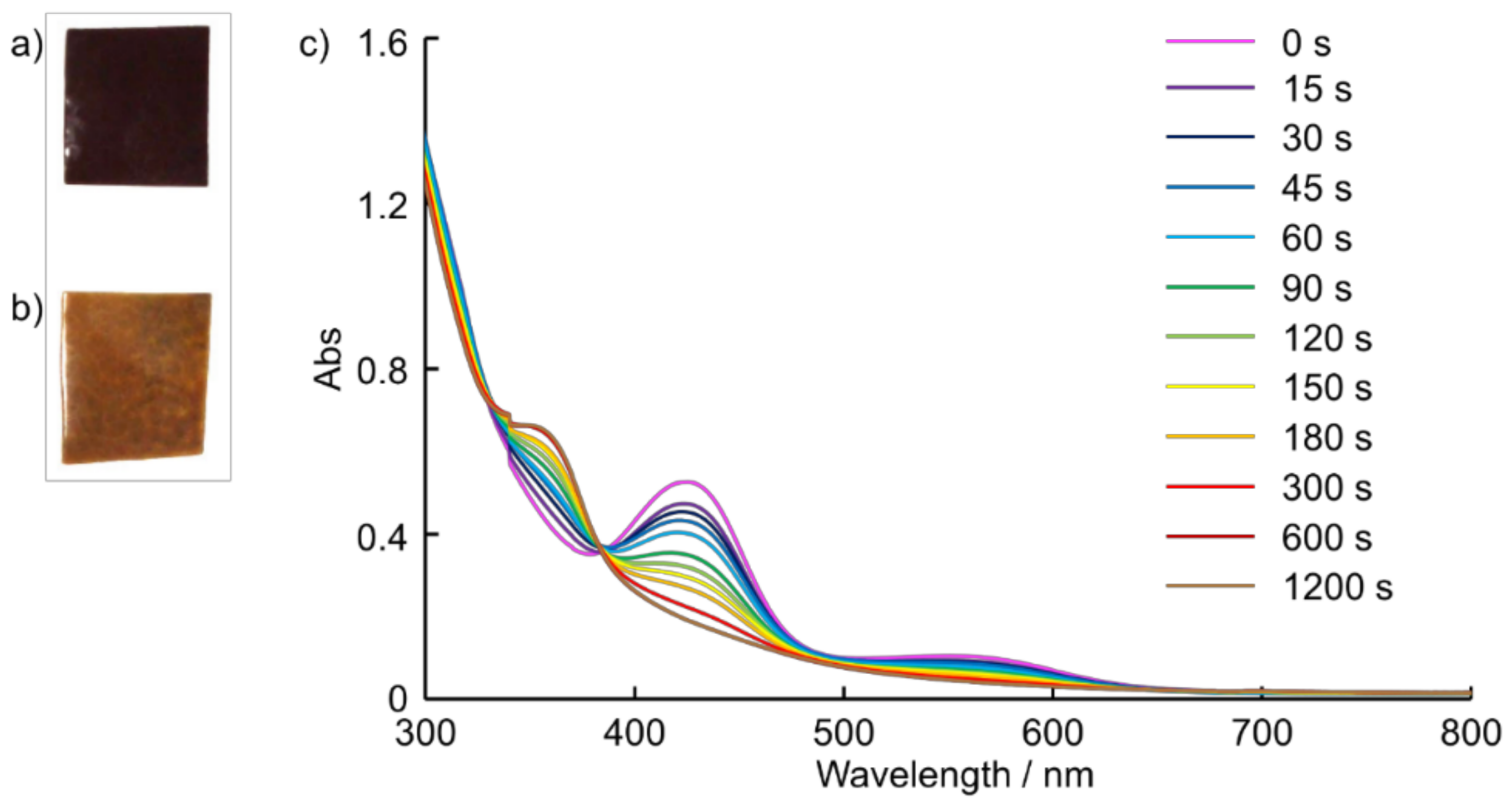
Photographs of (**a**) **1e**/polysiloxane self-standing composite film (thickness: 0.497 mm) and (**b**) **2e**/polysiloxane composite film, which was prepared by exposure of the **1e**/polysiloxane composite to CO gas. (**c**) UV-Vis spectral changes of the **1e**/polysiloxane self-standing composite film during exposure to CO gas.

To prepare a self-standing composite film, we used polysiloxane, which was synthesized by hydrolytic polycondensation of tetraethoxysilane (TEOS) in the presence of hydrogen chloride under a nitrogen flow, because of its high transparency, heat resistance, and gas permeability. Accordingly, self-standing composite films of **1c** and **1e** were prepared by a mixture of polysiloxane and **1c** or **1e**/THF solution to stand for 6 h at room temperature. The photographs of **1c**/polysiloxane and **1e**/polysiloxane self-standing composite film were shown in Figure 4a and Figure 5a, respectively. Both films show two main absorption peaks around 550 nm and 430 nm (Figure 4c and Figure 5c) that are almost the same absorption wavelength as **1c** and **1e** in a solution state. It is indicated that **1c** and **1e** are contained in polysiloxane without decomposition. After the composite films’ exposure to CO gas, these absorption peaks were immediately decreased without a peak shift. This result means that **1c** and **1e** were easily converted into **2c** and **2e** in the polysiloxane film during CO gas exposure. The reactivity of **1c** and **1e** composite films to CO gas seems to not be different from **1a**/polysiloxane composite film [23]. Unfortunately, the color change of **1c** and **1e** composite films during an exposure to CO gas were unclear compared with that of **1a** composite film by visual observation. We also noted that **1a** and polysiloxane make a uniform composite film [23], though **1c** and **1e** seem to be aggregated in the polysiloxane films. This is because **1c** and **1e** become less soluble to polysiloxane compared with **1a**. A chloride ligand on benzene ring in **1c** and **1e** led to a decrease in solubility to a polysiloxane.

## 3. Materials and Methods

### 3.1. General

Unless otherwise indicated, all synthesis reactions were carried out under an argon atmosphere. All solvents were degassed with nitrogen before use. Tetrahydrofuran, dichloromethane, hexane, benzene, and ethanol were purchased from KANTO Chemicals. Tetrahydrofuran and benzene were distilled from Na and, dried over 4 Å molecular sieves. Dichloromethane and hexane were dried over 4 Å molecular sieves. Ethanol was dried over 3 Å molecular sieves. 1,2-Dichloroethane and Hydrochloric acid (6 mol/L) were purchased from Wako Pure Chemical Industries, Ltd. (Osaka, Japan). 1,2-Dichloroethane was dried over 4 Å molecular sieves. Tetraethoxysilane was purchased from Shin-Etsu Chemical Co., Ltd. (Tokyo, Japan).

[(*η*^6^-C_6_Me_6_)RuCl_2_]_2_ was prepared according to the literature method [29].

All NMR spectra were recorded on a JEOL ECP-500 (500 MHz) spectrometer (Tokyo, Japan). ^1^H NMR (500 MHz) and ^13^C{^1^H} NMR (126 MHz) spectra were recorded using tetramethylsilane as an internal standard. ^19^F{^1^H} NMR (470 MHz) spectra were recorded using trifluoroacetic acid as an external standard. IR spectra were recorded with a JASCO FT-IR 6100 spectrometer (Tokyo, Japan), and UV-Vis-near infrared (NIR) spectra were recorded with a JASCO V670 spectrometer (Tokyo, Japan). Melting points were recorded using a Bibby Stuart Scientific SMP3 instrument (Staffordshire, UK); all melting points reported are uncorrected. HR-ESI mass spectra were recorded on a JEOL JMS-T100CS AccuTOF CS spectrometer (Tokyo, Japan).

All compounds were successfully identified by NMR spectroscopy, high-resolution mass spectroscopy, and single crystal X-ray spectroscopy. Unfortunately, trace impurities and solvent were observed in some NMR spectra after purification (see NMR spectra in supporting information). Therefore, we have not been carried out the elemental analysis.

### 3.2. X-ray Crystallography

Crystal data were collected using a Bruker AXS SMART APEX CCD X-ray diffractometer (Kanagawa, Japan) equipped with monochromatic Mo Kα radiation (0.7107 Å). Empirical absorption corrections using equivalent reflections and Lorentzian polarization corrections were performed using the SADABS program [30]. All data were collected with SMART and Bruker SAINTPLUS (Version 6.45) (Kanagawa, Japan) software packages. The structures were solved using the SHELXA-97 program [31]. and refined against *F*^2^ by using SHELEXL-97 [32]. We note that **1c**, **1d**, **2b**, **2c**, and **2e** show high *R*1-values (>8.0%) (Tables S1 and S2). This is because the rotation of the C_6_Me_6_ ring and rage anisotropic temperature factor of crystal solvents.

### 3.3. Synthesis

#### 3.3.1. General Procedure for the Synthesis of Dihalogen-Substituted Benzendithiols **3b**–**e**

The dihalogen-substituted benzendithiols were prepared with a slight modification of our previously reported method [24] using iron(II) chloride tetrahydrate instead of iron powder. The yield of these compounds was increased; **3b** (75%), **3c** (83%), **3d** (86%), **3e** (98%).

#### 3.3.2. General Procedure for the Synthesis of Ruthenium Dithiolene Complexes **1b**–**e**

A THF solution (20 mL) of dihalogen-substituted benzendithiol (1.0 mmol) and [(*η*^6^-C_6_Me_6_)RuCl_2_]_2_ (334 mg, 1.0 mmol) was stirred for 2 h at room temperature. The reaction mixture was evaporated and the crude product was purified by alumina column chromatography using dichloromethane as the eluent. The first fraction was collected and evaporated to yield the ruthenium dithiolene complex as a powder.

*(η^6^-C_6_Me_6_)Ru(S_2_C_6_F_2_H_2_)* (**1b**). The powder was recrystallized from dichloromethane/hexane at −30 °C to give the deep purple single crystals suitable for X-ray crystallographic analysis. Yield: 61%. ^1^H NMR (500 MHz, CDCl_3_) *δ* 2.36 (s, 18H, C_6_Me_6_), 6.74 (t, *J* = 6.5 Hz, 2H, S_2_C_6_F_2_H_2_). ^13^C{^1^H} NMR (126 MHz, CDCl_3_) *δ* 17.7 (s, C_6_Me_6_), 92.1 (s, C_6_Me_6_), 107.8 (dd, *J =* 19, 12 Hz), 143.9 (dd, *J* = 14, 11 Hz), 156.1 (d, ^2^*J*_C-F_ = 241 Hz, CF). ^19^F{^1^H} NMR (470 MHz, CDCl_3_) *δ* –88.8 (s). LRMS (ESI-TOF, positive) *m*/*z* 462 [M + Na]^+^. HRMS (ESI-TOF, positive), *m*/*z* calcd for C_18_H_20_F_2_Ru_1_S_2_Na_1_ 462.99157 [M + Na]^+^; found 462.99205.

*(η^6^-C_6_Me_6_)Ru(S_2_C_6_Cl_2_H_2_)* (**1c**). The powder was recrystallized from dichloromethane/hexane at −30 °C to give the deep purple single crystals. Yield: 63%. ^1^H NMR (500 MHz, CDCl_3_) *δ* 2.37 (s, 18H, C_6_Me_6_), 7.18 (s, 2H, S_2_C_6_Cl_2_H_2_). ^13^C{^1^H} NMR (126 MHz, CDCl_3_) *δ* 17.7 (s, C_6_Me_6_), 92.3 (s, C_6_Me_6_), 123.3 (s), 131.8 (s), 152.9 (s). HRMS (ESI-TOF, positive), *m*/*z* calcd for C_18_H_20_Cl_2_Ru_1_S_2_Na_1_ 494.93247 [M + Na]^+^; found 494.93471.

*(η^6^-C_6_Me_6_)Ru(S_2_C_6_Br_2_H_2_)* (**1d**). The powder was recrystallized from dichloromethane/hexane at room temperature to give the deep purple single crystals. Yield: 59%. ^1^H NMR (500 MHz, CDCl_3_) *δ* 2.37 (s, 18H, C_6_Me_6_), 7.29 (s, 2H, S_2_C_6_Br_2_H_2_). ^13^C{^1^H} NMR (126 MHz, CDCl_3_) *δ* 17.8 (s, C_6_Me_6_), 92.4 (s, C_6_Me_6_), 123.3 (s), 127.0 (s), 154.3 (s). HRMS (ESI-TOF, positive), *m*/*z* calcd for C_18_H_20_Br_2_Ru_1_S_2_Na_1_ 582.83144 [M + Na]^+^; found 582.83214.

*(η^6^-C_6_Me_6_)Ru(S_2_C_6_H_2_Cl_2_)* (**1e**). The powder was recrystallized from dichloromethane/hexane at −30 °C to give the deep orange single crystals. Yield: 47%. ^1^H NMR (500 MHz, CDCl_3_) *δ* 2.34 (s, 18H, C_6_Me_6_), 7.90 (s, 2H, S_2_C_6_Br_2_H_2_). ^13^C{^1^H} NMR (126 MHz, CDCl_3_) *δ* 17.7 (s, C_6_Me_6_), 91.8 (s, C_6_Me_6_), 126.0 (s), 130.1 (s), 153.8 (s). HRMS (ESI-TOF, positive), *m*/*z* calcd for C_18_H_20_Cl_2_Ru_1_S_2_Na_1_ 494.93247 [M + Na]^+^; found 494.93286.

#### 3.3.3. General Procedure for the Reaction of Ruthenium Carbonyl Complexes **2b**–**e**

A benzene solution (5 mL) of ruthenium dithiolene complex (0.10 mmol) was bubbled with CO gas for 10 s and stirred at room temperature for 24 h under a CO atmosphere. The precipitation was collected by filtration and washed with hexane. The product was dried under reduced pressure to give the CO addition product.

*(η^6^-C_6_Me_6_)Ru(S_2_C_6_F_2_H_2_)(CO)* (**2b**). The product was recrystallized from dichloromethane/hexane at room temperature to give the deep purple single crystals suitable for X-ray crystallographic analysis. Yield: 71%. ^1^H NMR (500 MHz, CDCl_3_) *δ* 2.18 (s, 18H, C_6_Me_6_), 6.43 (dd, *J* = 6.5, 6.5 Hz, 2H, S_2_C_6_F_2_H_2_). ^13^C{^1^H} NMR (126 MHz, CDCl_3_) *δ* 16.2 (s, C_6_Me_6_), 108.4 (dd, *J* = 20, 12 Hz), 109.8 (s, C_6_Me_6_), 157.1 (d, ^2^*J*_C-F_ = 237 Hz, CF), 195.6 (s, CO) (the signal attributed to benzendithiolate ligand was not appeared). ^19^F{^1^H} NMR (470 MHz, CDCl_3_) *δ* –88.5 (s). HRMS (ESI-TOF, positive), *m*/*z* calcd for C_19_H_20_F_2_Ru_1_O_1_S_2_Na_1_ 490.98648 [M + Na]^+^; found 490.98629.

*(η^6^-C_6_Me_6_)Ru(S_2_C_6_Cl_2_H_2_)(CO)* (**2c**). The powder was recrystallized from dichloromethane/hexane at –30 °C to give the deep orange single crystals. Yield: 40%. ^1^H NMR (500 MHz, CDCl_3_) *δ* 2.18 (s, 18H, C_6_Me_6_), 6.76 (s, 2H, S_2_C_6_Cl_2_H_2_). ^13^C{^1^H} NMR (126 MHz, CDCl_3_) *δ* 16.1 (s, C_6_Me_6_), 110.1 (s, C_6_Me_6_), 123.5 (s), 130.3 (s), 145.0 (s), 195.7 (s, CO). HRMS (ESI-TOF, positive), *m*/*z* calcd for C_19_H_20_Cl_2_Ru_1_O_1_S_2_Na_1_ 522.92738 [M + Na]^+^; found 522.92756.

*(η^6^-C_6_Me_6_)Ru(S_2_C_6_Br_2_H_2_)(CO)* (**2d**). The powder was recrystallized from dichlorobenzene/hexane at –30 °C to give the deep orange single crystals. Yield: 75%. ^1^H NMR (500 MHz, CDCl_3_) δ 2.17 (s, 18H, C_6_Me_6_), 6.84 (s, 2H, S_2_C_6_Cl_2_H_2_). ^13^C{^1^H} NMR (126 MHz, CDCl_3_) *δ* 16.1 (s, C_6_Me_6_), 110.3 (s, C_6_Me_6_), 121.2 (s), 127.0 (s), 146.4 (s), 195.7 (s, CO). HRMS (ESI-TOF, positive), *m*/*z* calcd for C_19_H_20_Br_2_Ru_1_O_1_S_2_Na_1_ 610.82635 [M + Na]^+^; found 610.82640.

*(η^6^-C_6_Me_6_)Ru(S_2_C_6_H_2_Cl_2_)(CO)* (**2e**). The powder was recrystallized from dichloroethane/hexane at room tempaerature to give the red single crystals. Yield: 80%. ^1^H NMR (500 MHz, CDCl_3_) *δ* 2.14 (s, 18H, C_6_Me_6_), 7.20 (s, 2H, S_2_C_6_Cl_2_H_2_). ^13^C{^1^H} NMR (126 MHz, CDCl_3_) *δ* 16.1 (s, C_6_Me_6_), 109.8 (s, C_6_Me_6_), 124.3 (s), 127.4 (s), 145.1 (s), 196.1 (s, CO). HRMS (ESI-TOF, positive), *m*/*z* calcd for C_19_H_20_Cl_2_Ru_1_O_1_S_2_Na_1_ 522.92738 [M + Na]^+^; found 522.92719.

#### 3.3.4. Preparation of Polysiloxane

An aqueous HCl solution [mixture of 6 mol/L HCl aq. (1.91 g) and H_2_O (1.55 g)] was added to a mixture of tetraethoxysilane (20.8 g, 0.100 mol) and ethanol (9.21 g, 0.200 mol) in a 200 mL four-necked flask equipped with a stirrer and N_2_ gas inlet and outlet tubes at 0 °C in a H_2_O/tetraethoxysilane molar ratio of 1.70. The mixture was stirred at 0 °C for 10 min, then stirred at room temperature for 10 min and then heated at 80 °C for 4 h with stirring at a rate of 150 rpm under a N_2_ stream (360 mL/min flow rate). A 20 wt % solution was prepared by dissolving polysiloxane in THF, and polysiloxane was obtained as a colorless viscous liquid (8.71 g, *M*_w_ = 6600, *M*_w_/*M*_n_ = 1.3).

#### 3.3.5. Preparation of Self-Standing Composite Film

A mixture (10 g) of 20 wt % THF solution of polysiloxane and ruthenium dithiolene complex (4 or 10 mg) was stirred at room temperature for 1 h. An aliquot of the solution was cast onto a polytetrafluoroethylene petri dish with a 55 mm diameter, and left to stand at room temperature for 6 h to form the self-standing film.

#### 3.3.6. Reaction of a Composite Film with CO Gas

A 1 cm square of the composite film was placed in the 100 mL recovery flask, and still for a prescribed time under CO atmosphere at room temperature.

## 4. Conclusions

In summary, novel ruthenium dithiolene complexes **1b**–**e** were successfully synthesized from dihalogen-substituted benzendithiols **3b**–**e** using in an almost similar manner to a general method for synthesis of half-sandwich type metalladithiolene complexes. The carbonyl ligand adduct complexes **2b**–**e** were obtained by the reaction of THF solution of **1b**–**e** under CO gas. The yield of dihalo-substituted complexes **1b**–**e** is low compared with that of **1a** because of the low electron density of ruthenium metal center compared with non-substituted complex **1a**. Ruthenium complexes **1b**–**e** show two strong absorption peaks around 550 and 420 nm. After exposure to CO gas, these absorption peaks were immediately decreased without a peak shift. Decreases of absorption were caused by the structural changes around the Ru atom from the vertical coordination between the C_6_Me_6_ ligand and dithiolene part to piano-stool geometry. **1c**/polysiloxane and **1e**/polysiloxane self-standing composite films were successfully prepared by a mixture of polysiloxane and **1c** or **1e**/THF solution. These films also reacted with CO gas and showed a similar color change in the solution state. This result means that **1c** and **1e** were easily converted into **2c** and **2e** in the polysiloxane film during CO gas exposure. The substituent groups on the benzene ring of benzendithiolate ligand are important to enable to fine adjustment of electron density of the metal center and to control the solubility to a polymer matrix. The knowledge gained from this study will contribute toward the development of advanced materials such as those containing metal complexes in a polymer matrix. The synthesis and reactivity of other types of ruthenium dithiolene complexes, which have electron donating substituent groups on the benzene ring of benzene dithiolate ligand, are currently under investigation.

## Figures and Tables

**Table 1:** C–O bond distance, Ru–C–O bond angles, and IR absorption of carbonyl group of **2a**–**e**.

	2a	2b	2c	2d	2e
C–O distance (Å)	1.133 (7)	1.122 (10)	1.150 (14)	1.09 (3) ^a^	1.22 (3) ^b^
Ru–C–O angle (°)	178.8 (6)	178.2 (8)	178.6 (10)	174 (2) ^a^	179 (2) ^b^
*ν*_CO_ (cm^−1^)	1952	1968	1976	1972	1979

^a^ Data is displayed for one of two molecules. ^b^ Data is displayed for one of three molecules.

## Supplementary Materials

Supplementary materials are available online. NMR, IR and UV-Vis-NIR spectra, crystal structures, and crystallographic data, TG–DTA curves, details of TD-DFT calculations, HOMO and LUMO orbitals. Deposition number CCDC 1588216 (for **1b**), 1588217 (for **1c**), 1588218 (for **1d**), 1588219 (for **1e**), 1588220 (for **2b**), 1588221 (for **2c**), 1588222 (for **2d**), and 1588223 (for **2e**) contain the supplementary crystallographic data for this paper. These data can be obtained free of charge via http://www.ccdc.cam.ac.uk/conts/retrieving.html (or from the Cambridge Crystallographic Data Centre, 12 Union Road, Cambridge, CB2 1EZ, UK; Fax: +44 1223 336033; e-mail: deposit@ccdc.cam.ac.uk).
