# Supplementary material for: Preparation of Ruthenium Dithiolene Complex/Polysiloxane Films and Their Responses to CO Gas
Source: Molecules. 2018 Apr 7;23(4):845. doi: 10.3390/molecules23040845 (PMC6017087; doi:10.3390/molecules23040845)
Supplement: Supplementary file 1 [file molecules-23-00845-s001.pdf]

Supporting Information Materials

for

Preparation of Ruthenium Dithiolene Complex/Polysiloxane Films and Response to CO Gas

Satoru Tsukada [1,*], Takuya Sagawa [2], Kazuki Yamamoto [2] and Takahiro Gunji [2]

[1]Advanced Materials Laboratory, Advanced Automotive Research Collaborative Laboratory, Graduate School of Engineering, Hiroshima University, 1-4-1 Kagamiyama, Higashi-Hiroshima, Hiroshima 739-8527, Japan

[2]Department of Pure and Applied Chemistry, Faculty of Science and Technology, Tokyo University of Science, 2641 Yamazaki, Noda, Chiba 278-8510, Japan; 7215705@ed.tus.ac.jp (T.S.); gunji@rs.noda.tus.ac.jp (T.G.)

* Correspondence: tsukada@hiroshima-u.ac.jp; Tel.: +81-82-424-7909

Contents

S2-14	NMR and mass spectral charts of **1b**-**1e** and **2b**-**2e**.
S15	IR spectra of **2a**-**2e**.
S16-18	Crystal and packing structures of **1b**, **1d**, **1e**, **2b**, **2d**, and **2e**.
S18-19	Summary of crystal data
S20-21	TG-DTA curves of **1c**, **1e**, **2c**, and **2e**.
S22	UV-Vis-NIR spectra of **1a**-**1e** and **2a**-**2e**.
S23-35	Computational details
S36	References

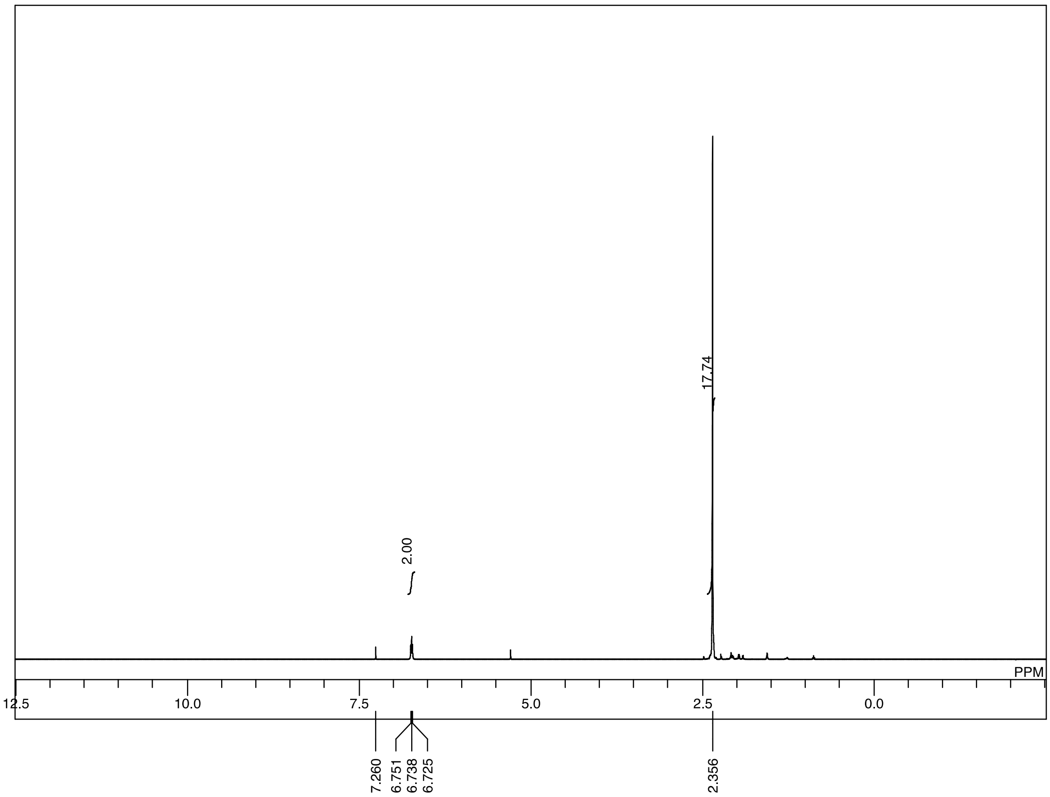

Figure S1. ^{1}H NMR spectrum of **1b** in $CDCl_3$.

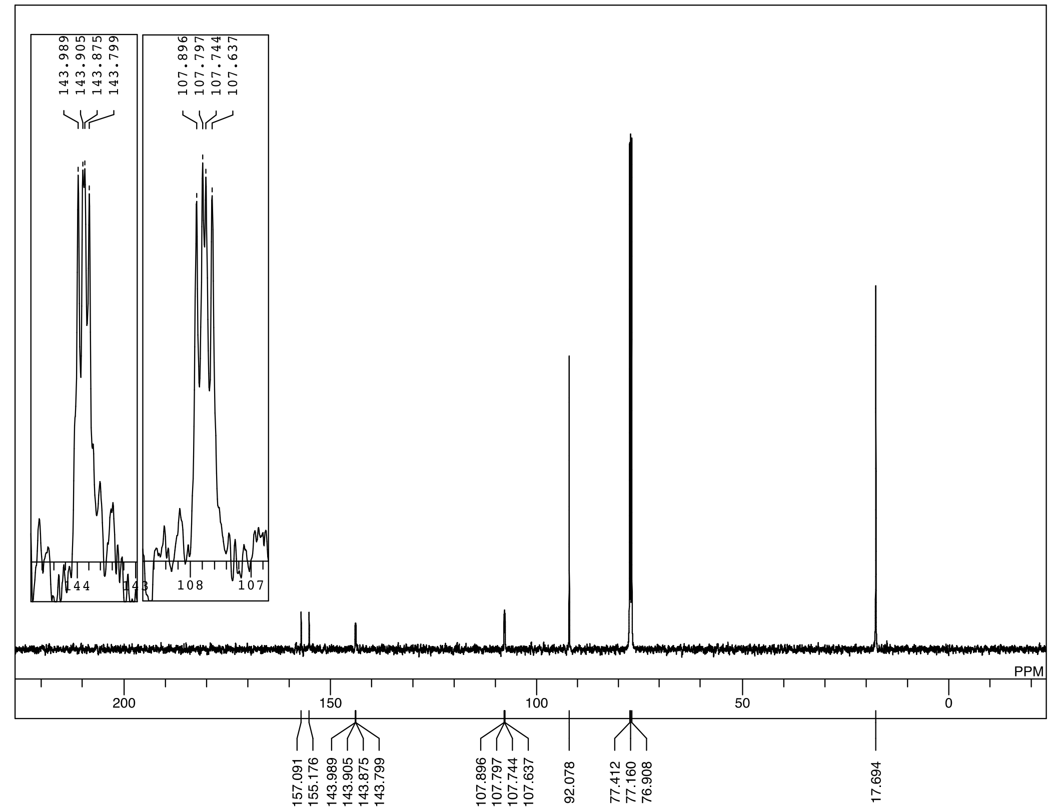

Figure S2. ^{13}C NMR spectrum of **1b** in $CDCl_3$.

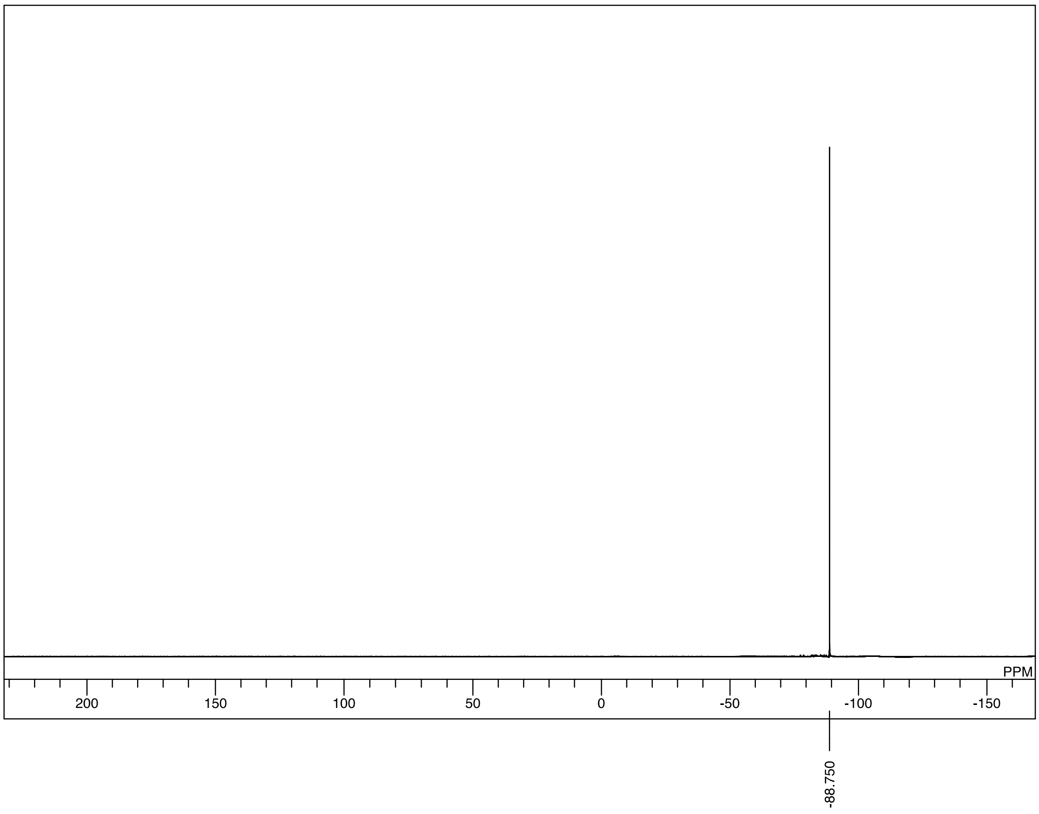

Figure S3. ^{19}F NMR spectrum of **1b** in $CDCl_3$.

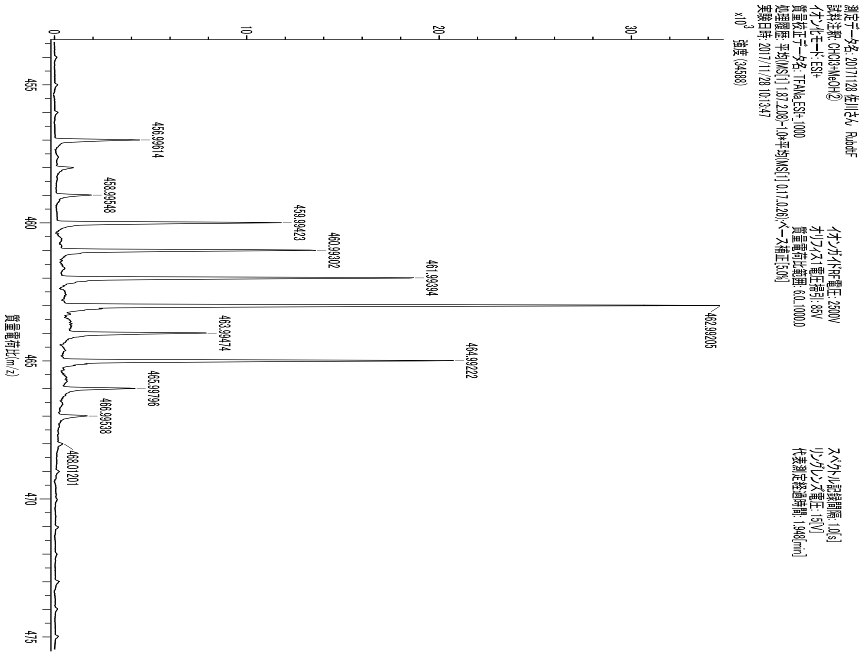

Figure S4. High resolution mass spectrum (ESI-TOF, positive) of **1b**.

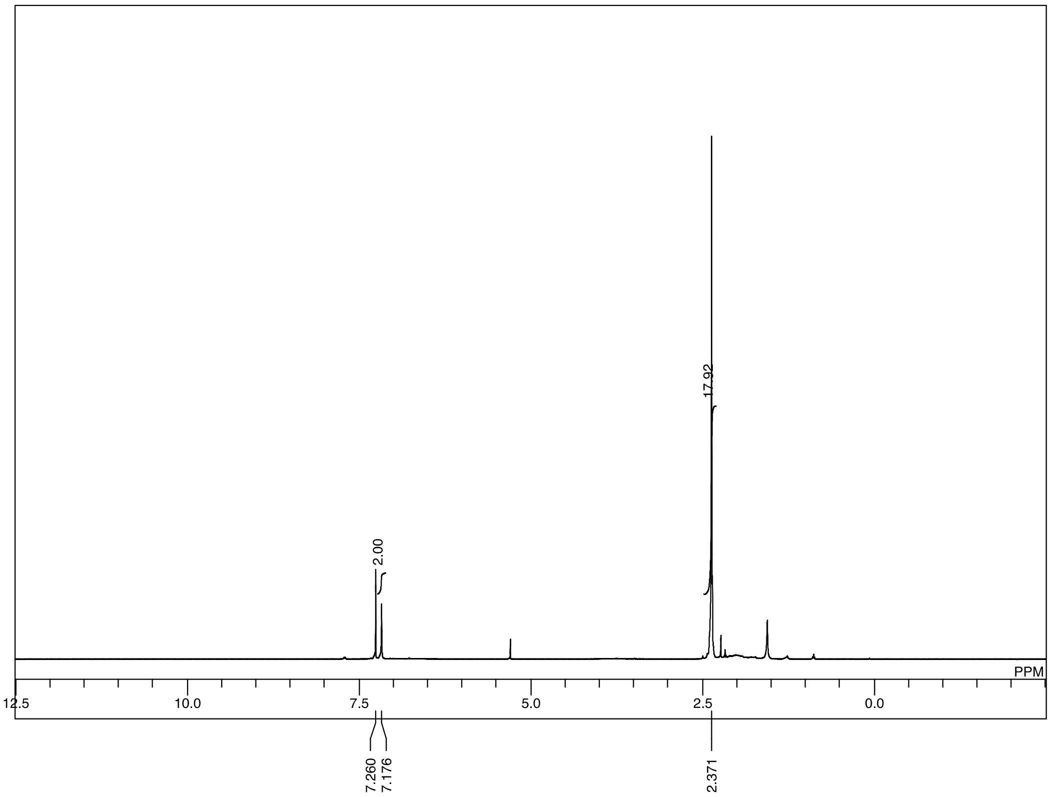

Figure S5. ^{1}H NMR spectrum of **1c** in $CDCl_3$.

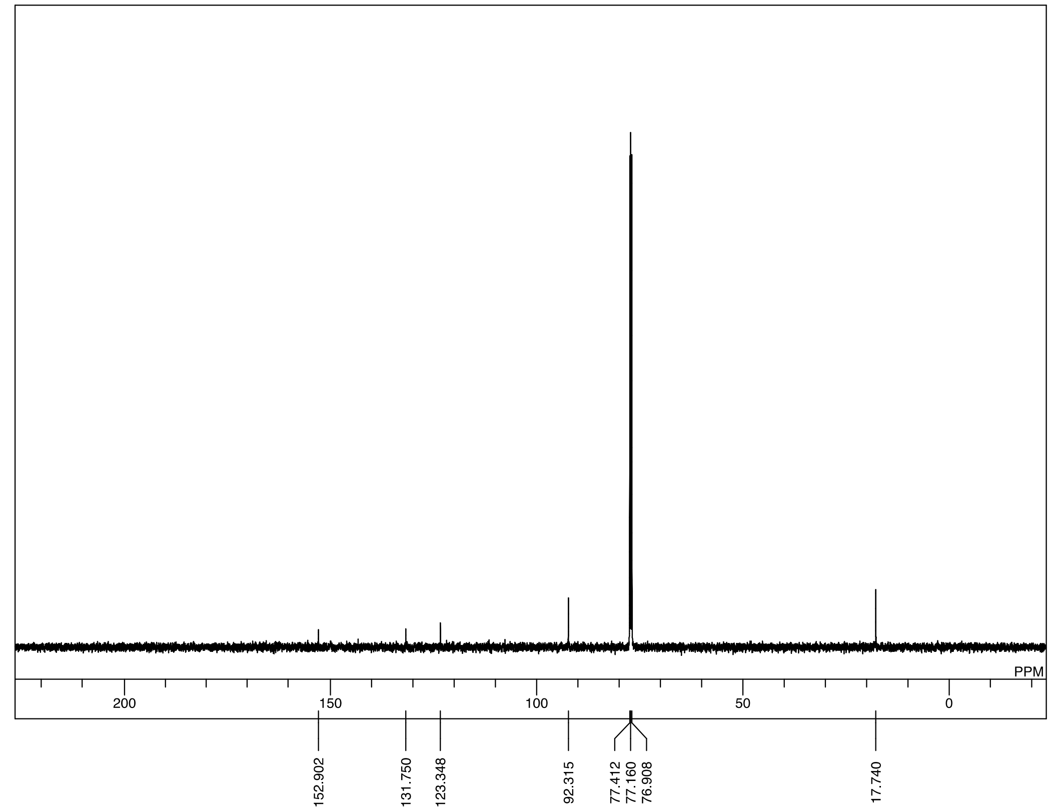

Figure S6. ^{13}C NMR spectrum of **1c** in $CDCl_3$.

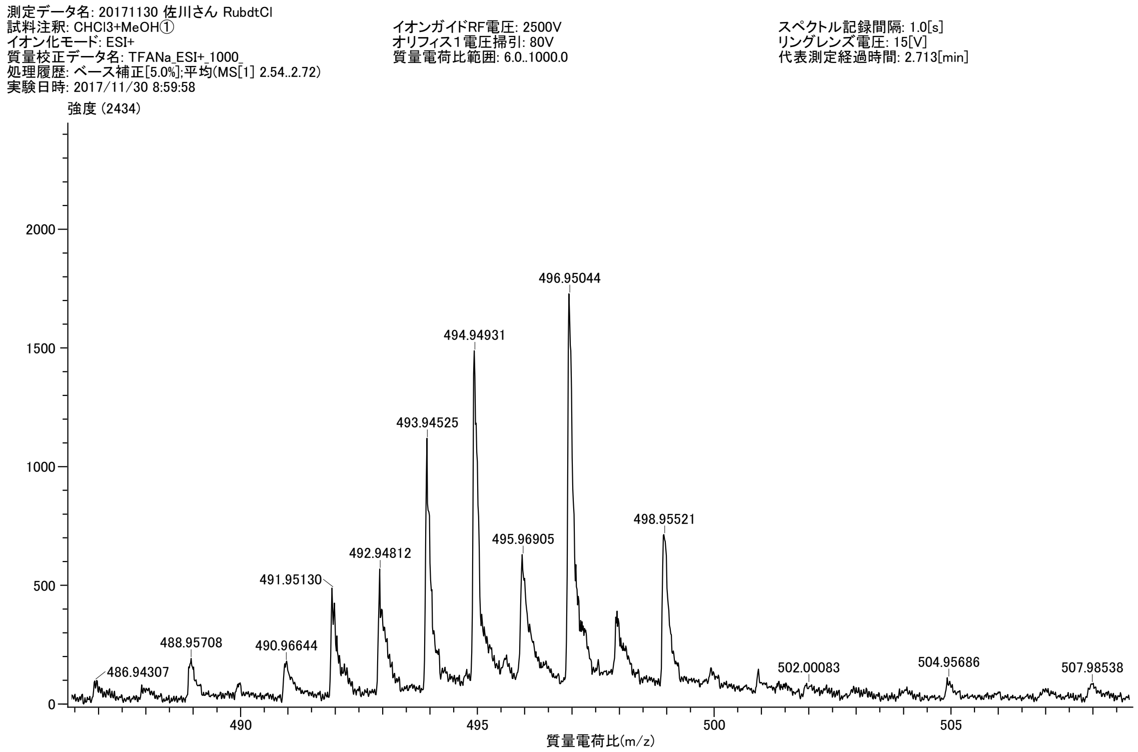

Figure S7. High resolution mass spectrum (ESI-TOF, positive) of **1c**.

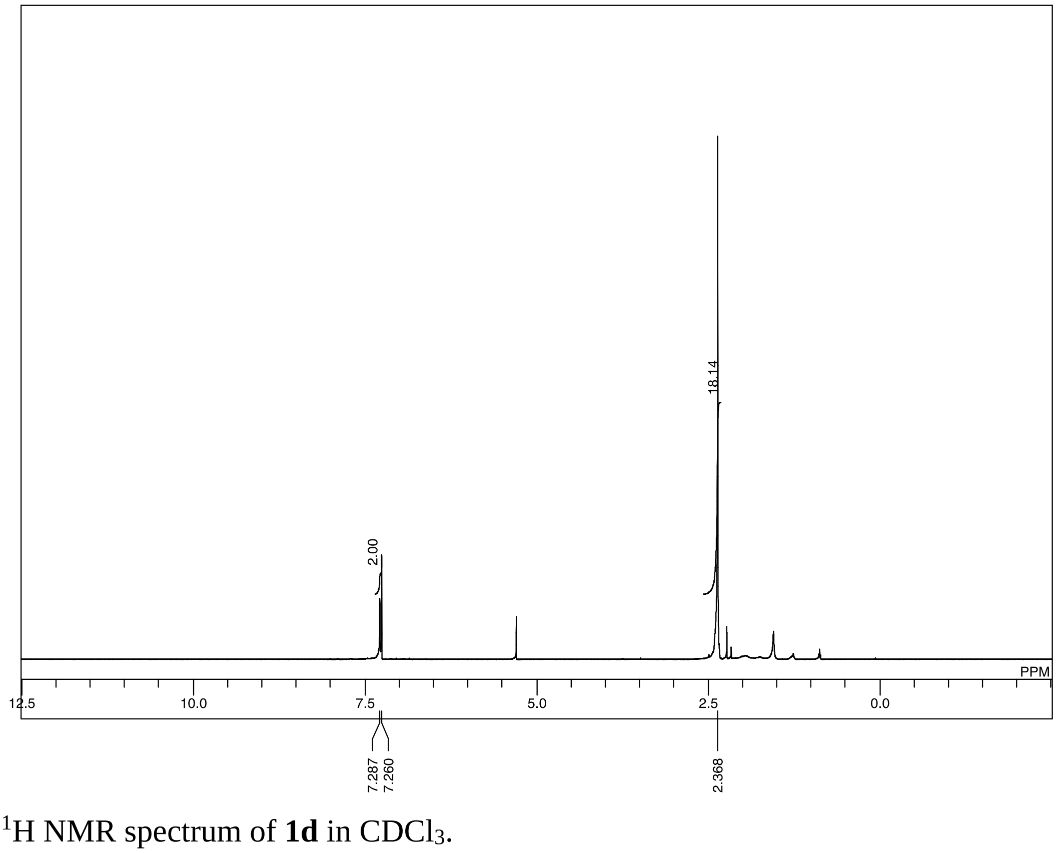

Figure S8. ^{1}H NMR spectrum of **1d** in $CDCl_3$.

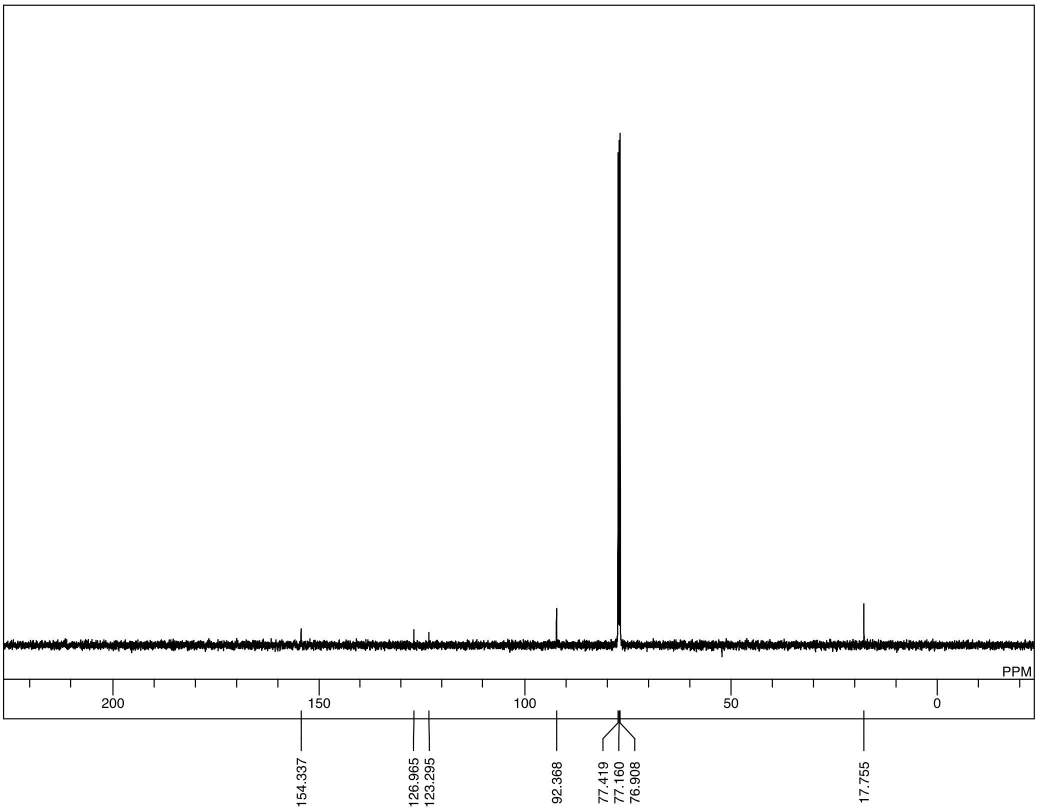

Figure S9. ^{13}C NMR spectrum of **1d** in $CDCl_3$.

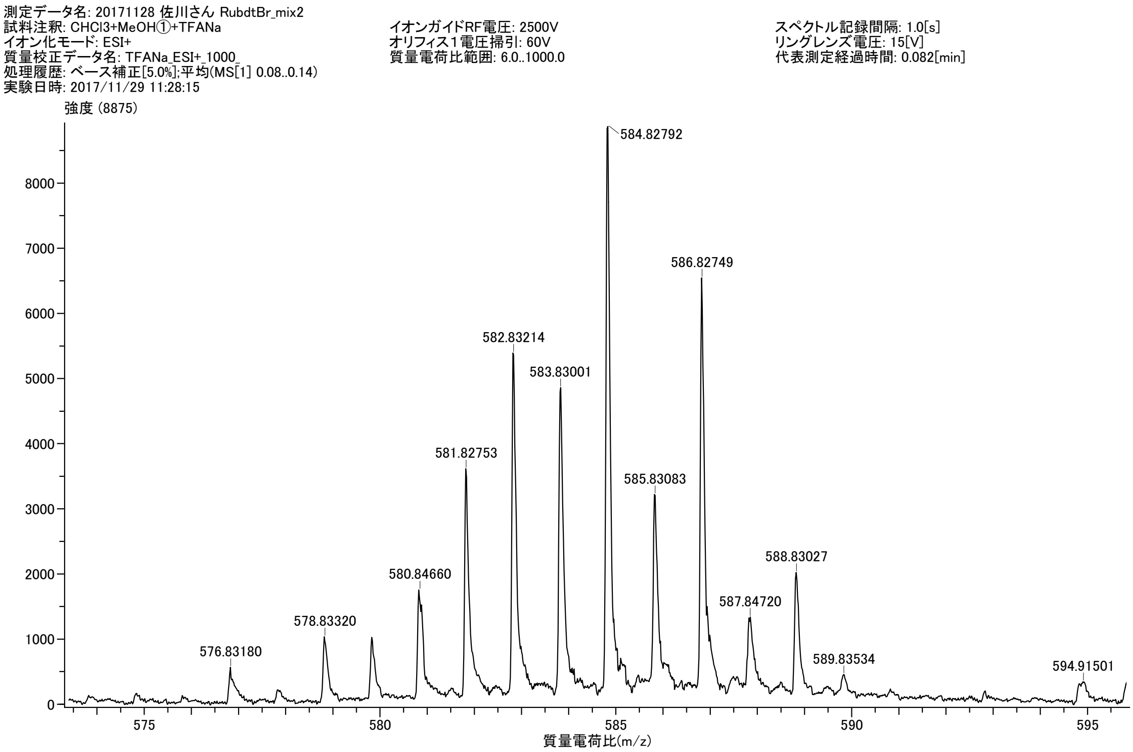

Figure S10. High resolution mass spectrum (ESI-TOF, positive) of **1d**.

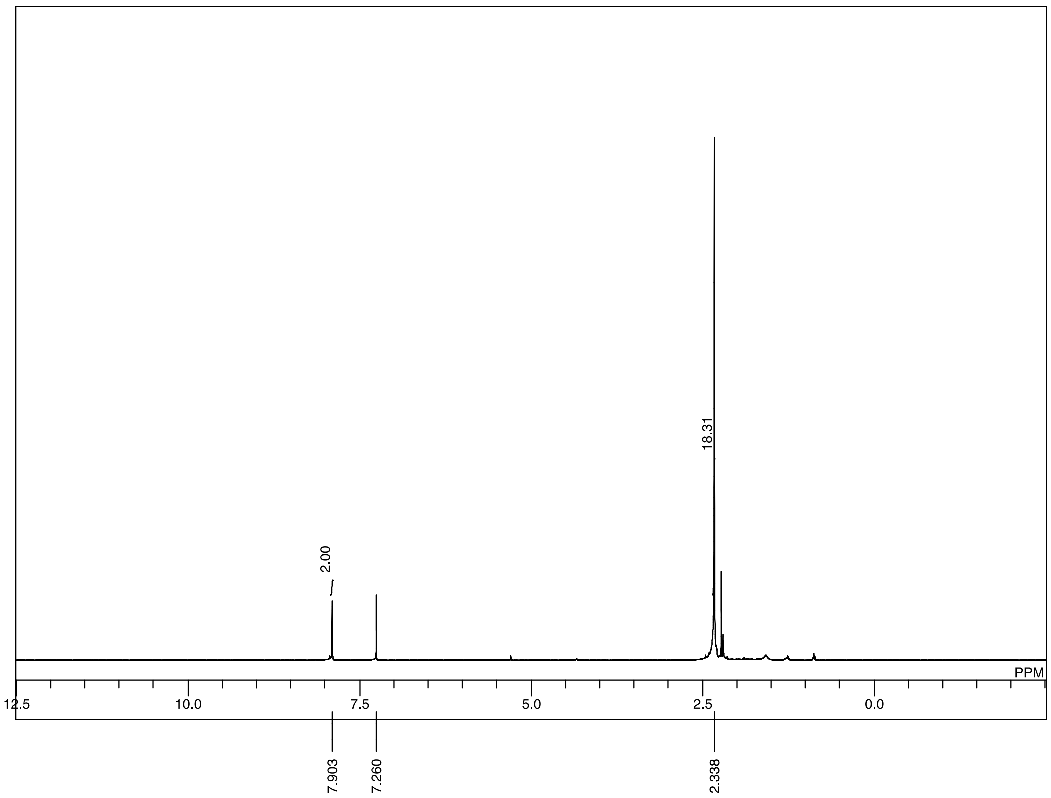

Figure S11. ^{1}H NMR spectrum of **1e** in $CDCl_3$.

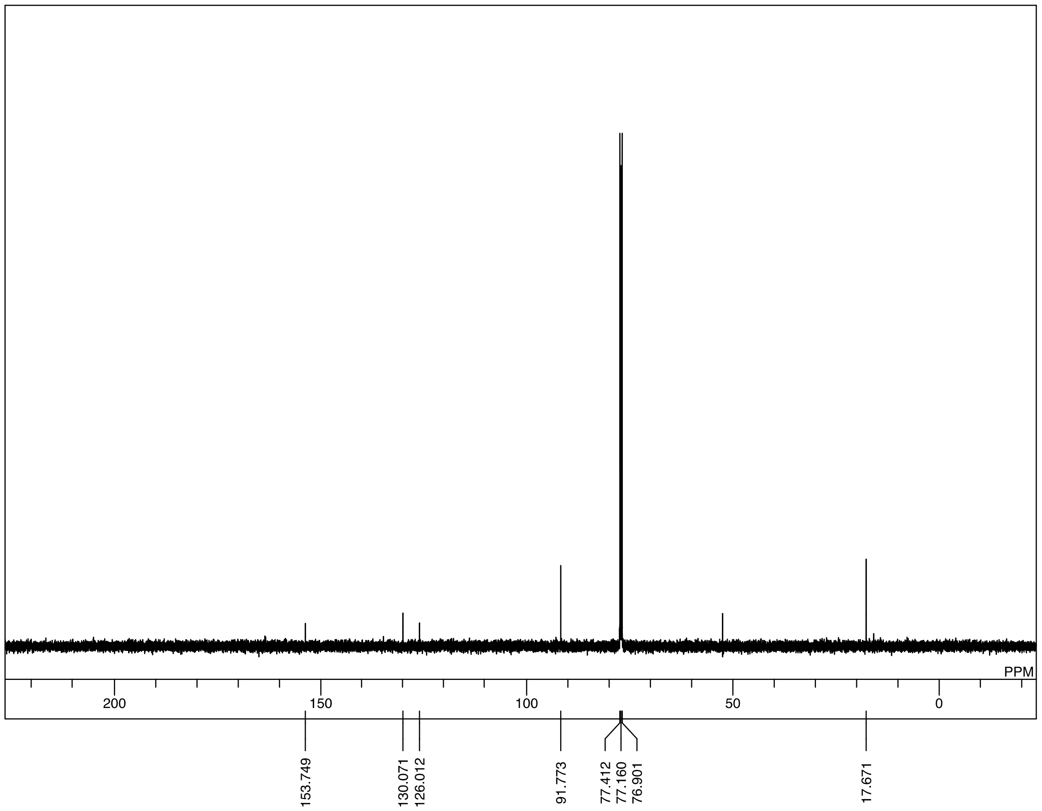

Figure S12. ^{13}C NMR spectrum of **1e** in $CDCl_3$.

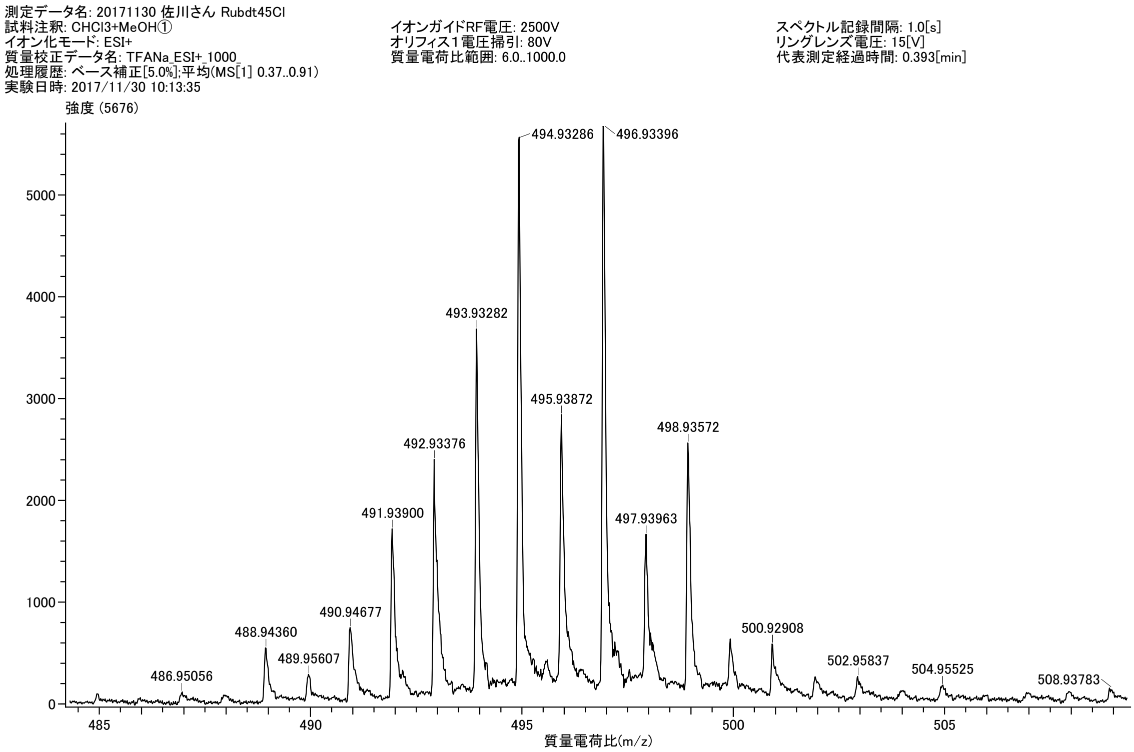

Figure S13. High resolution mass spectrum (ESI-TOF, positive) of **1e**.

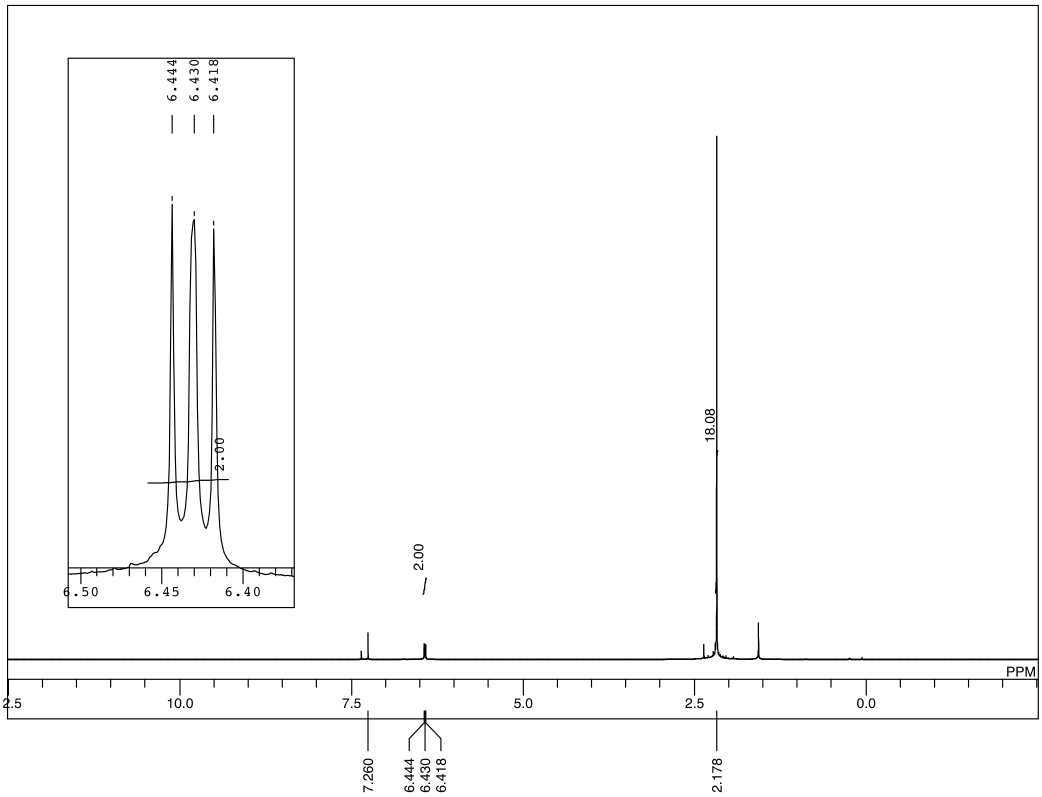

Figure S14. ^{1}H NMR spectrum of **2b** in $CDCl_3$.

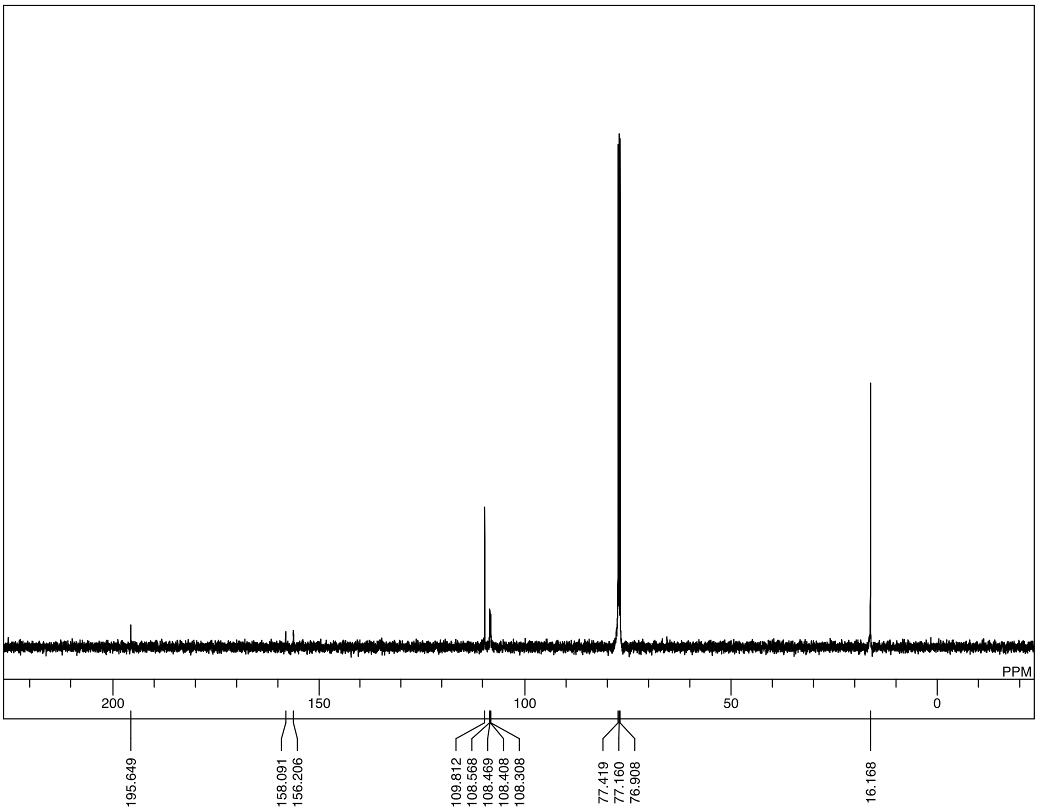

Figure S15. ^{13}C NMR spectrum of **2b** in $CDCl_3$.

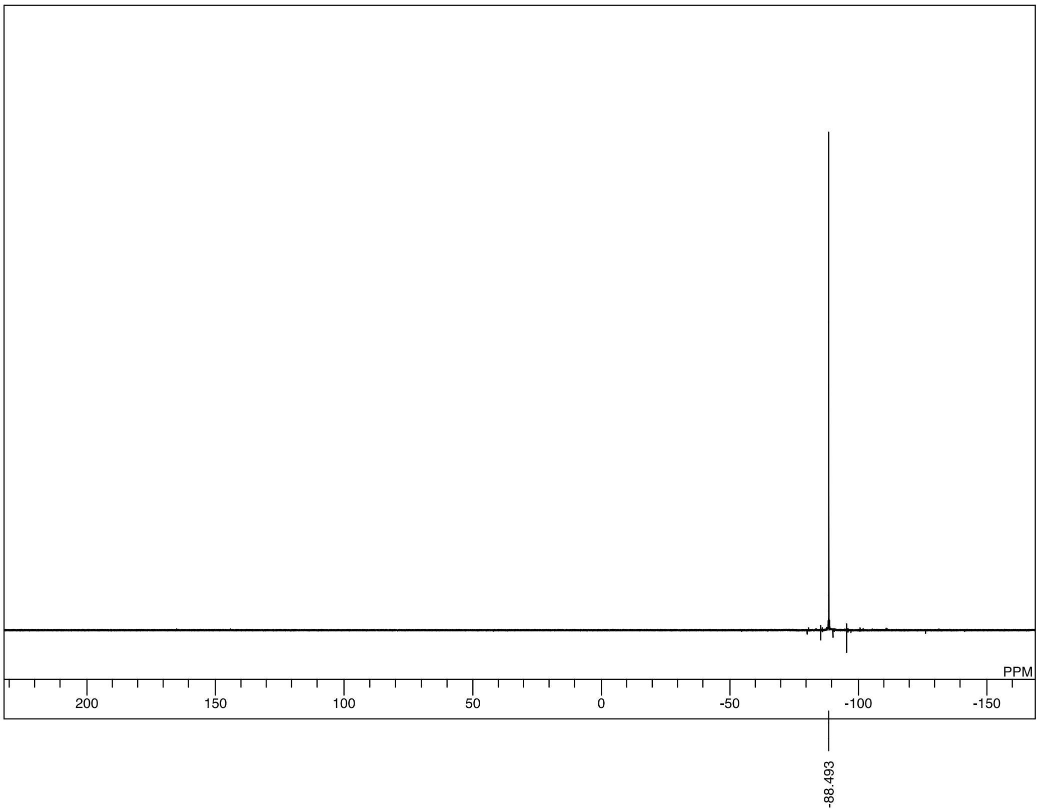

Figure S16. ^{19}F NMR spectrum of **2b** in $CDCl_3$.

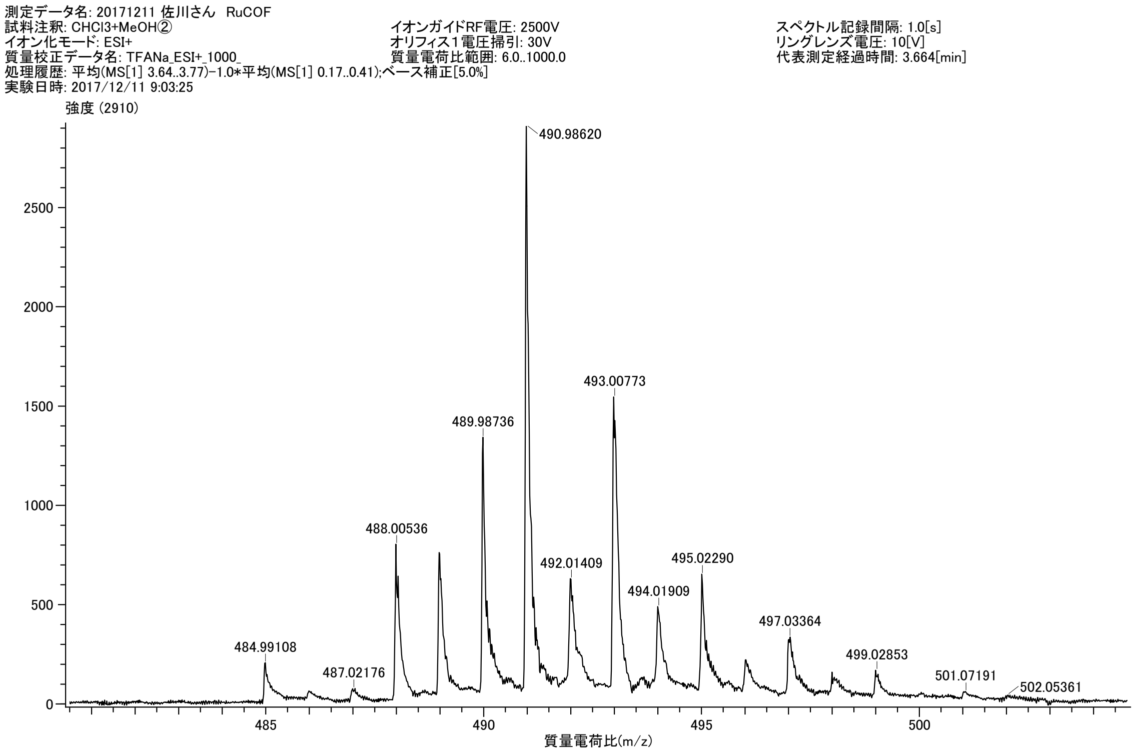

Figure S17. High resolution mass spectrum (ESI-TOF, positive) of **2b**.

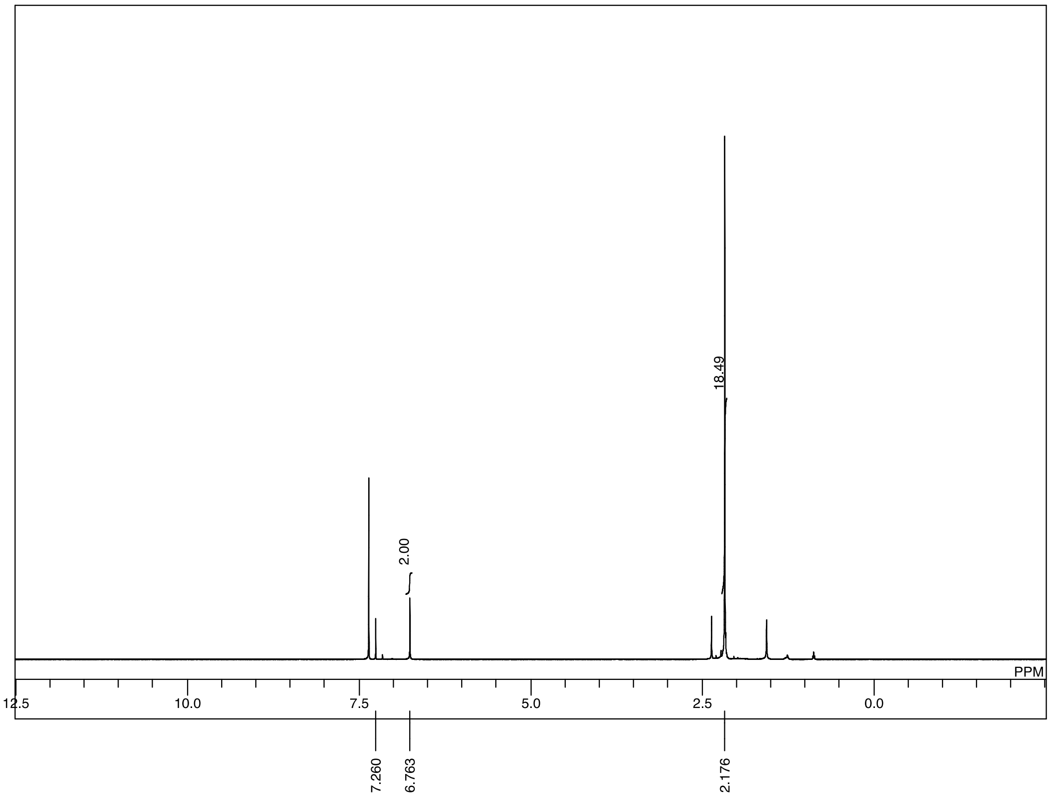

Figure S18. ^{1}H NMR spectrum of **2c** in $CDCl_3$.

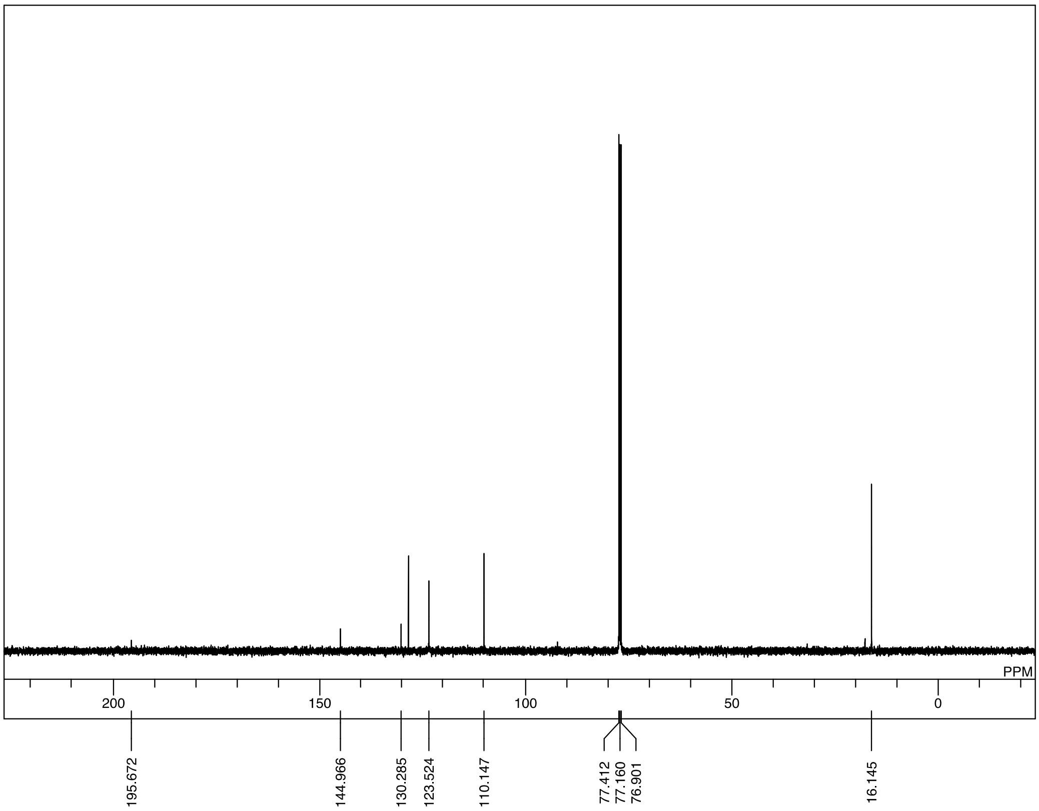

Figure S19. ^{13}C NMR spectrum of **2c** in $CDCl_3$.

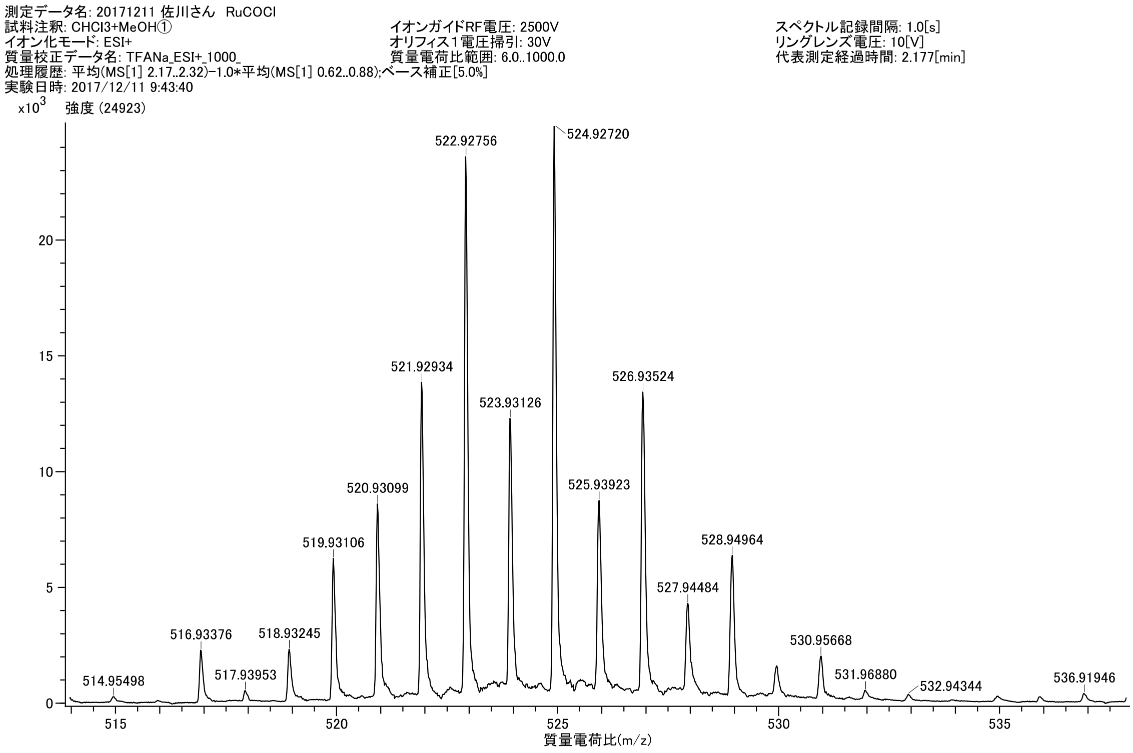

Figure S20. High resolution mass spectrum (ESI-TOF, positive) of **2c**.

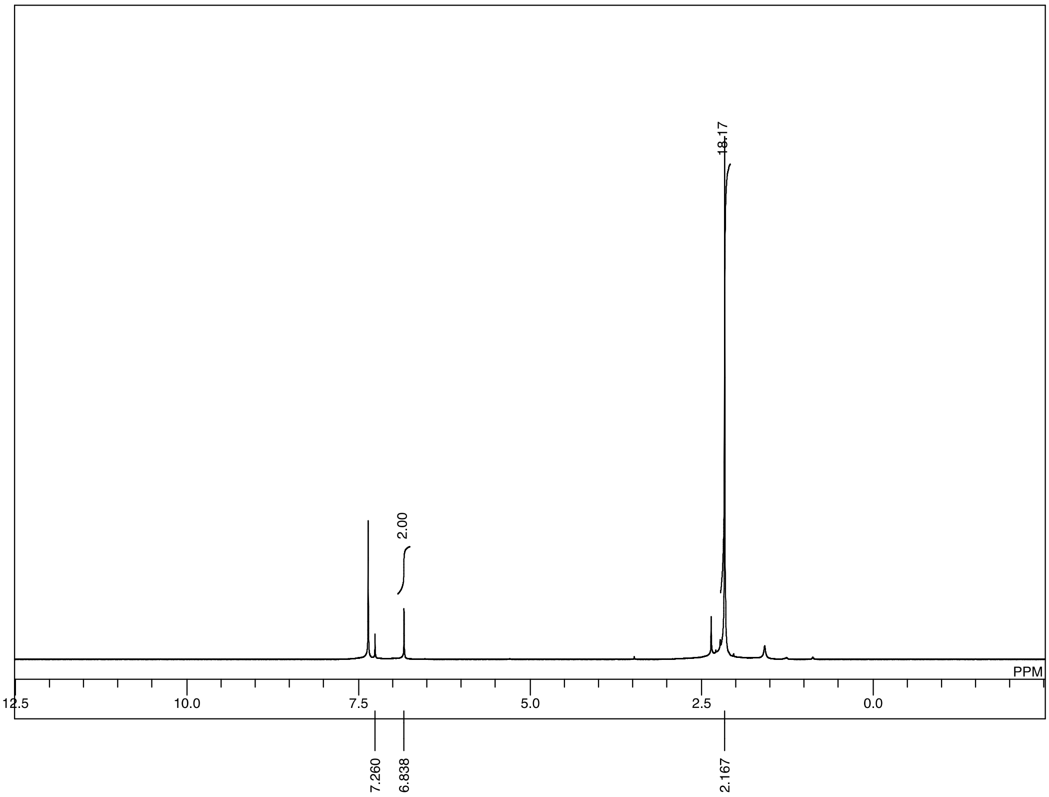

Figure S21. ^{1}H NMR spectrum of **2d** in $CDCl_3$.

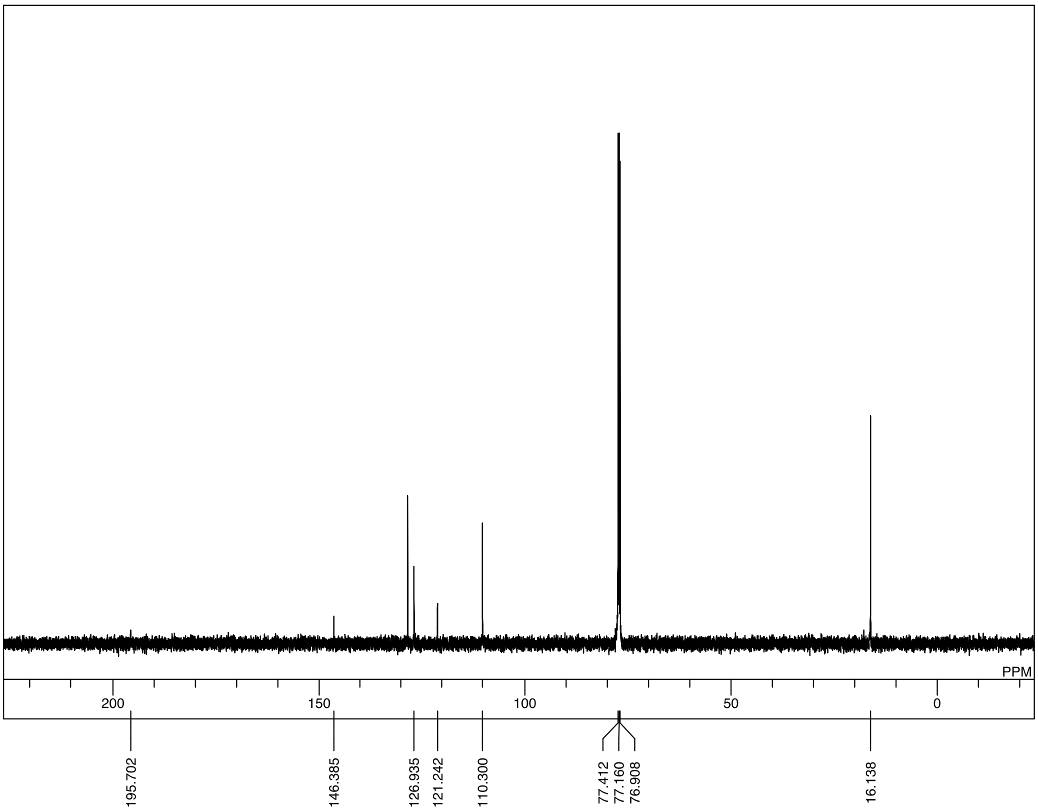

Figure S22. ^{13}C NMR spectrum of **2d** in $CDCl_3$.

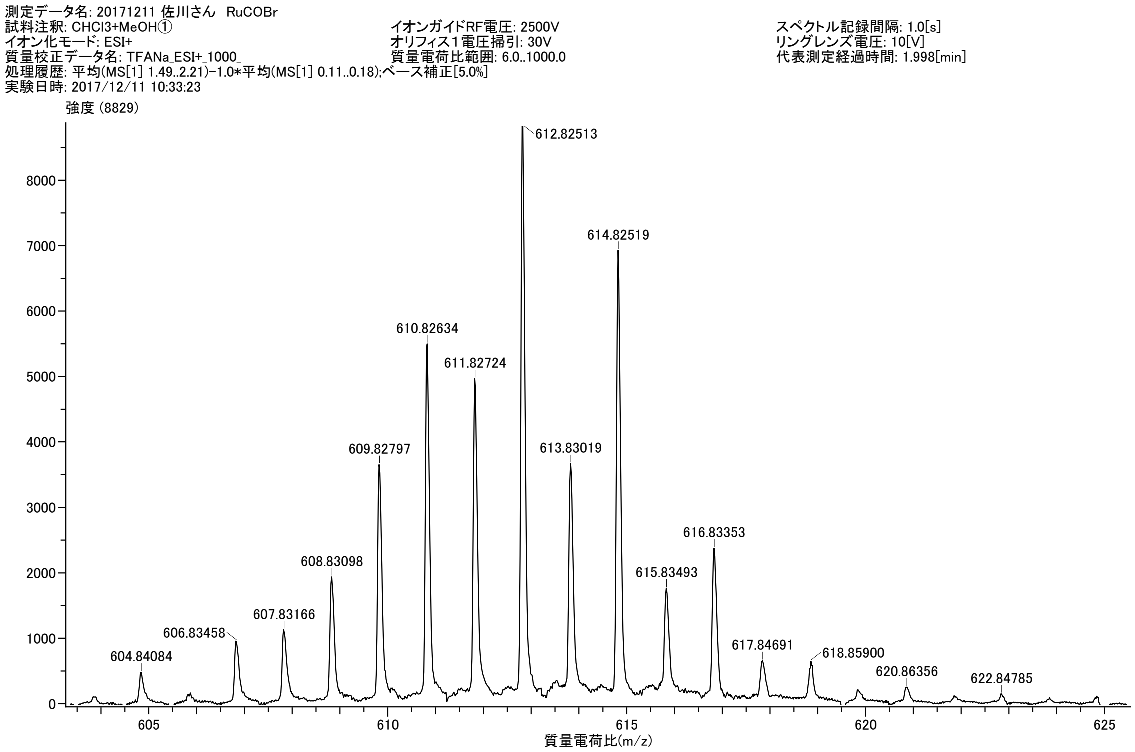

Figure S23. High resolution mass spectrum (ESI-TOF, positive) of **2d**.

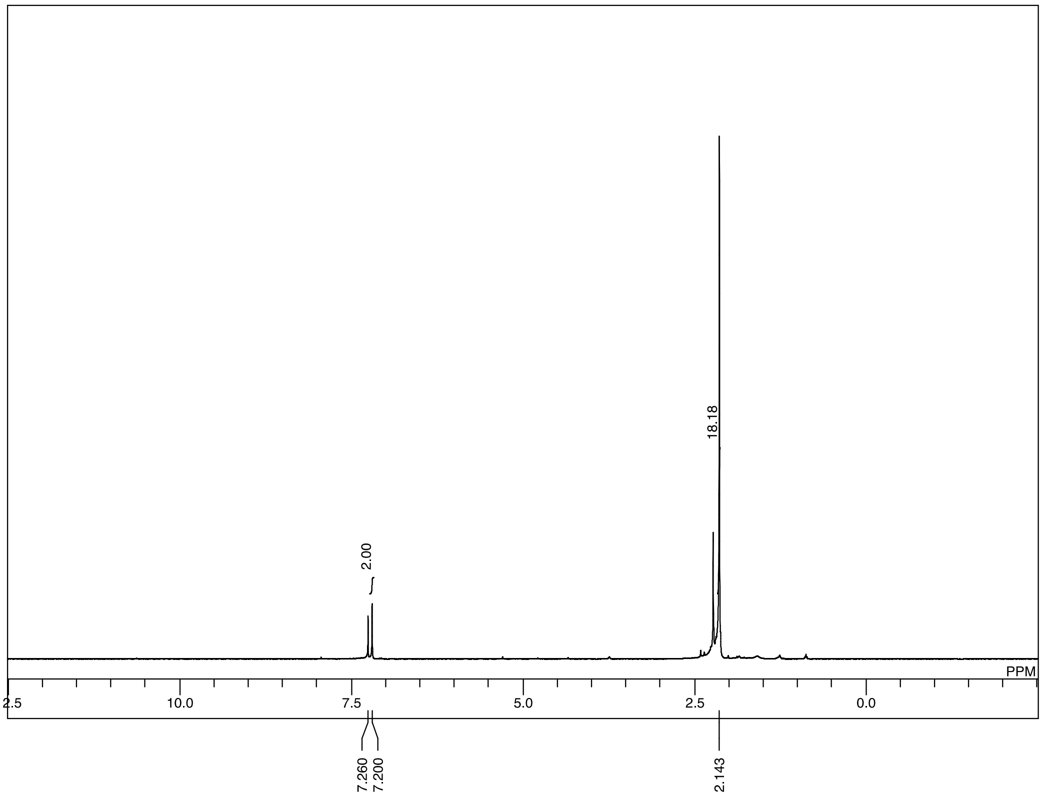

Figure S24. ^{1}H NMR spectrum of **2e** in $CDCl_3$.

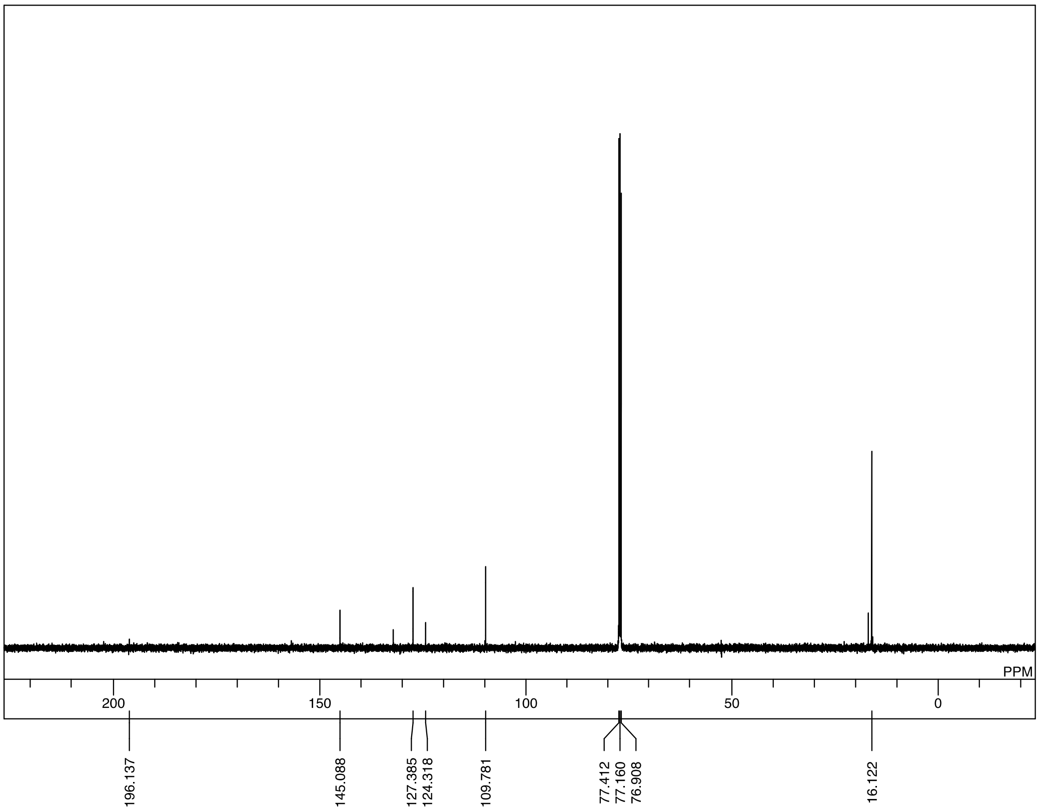

Figure S25. ^{13}C NMR spectrum of **2e** in $CDCl_3$.

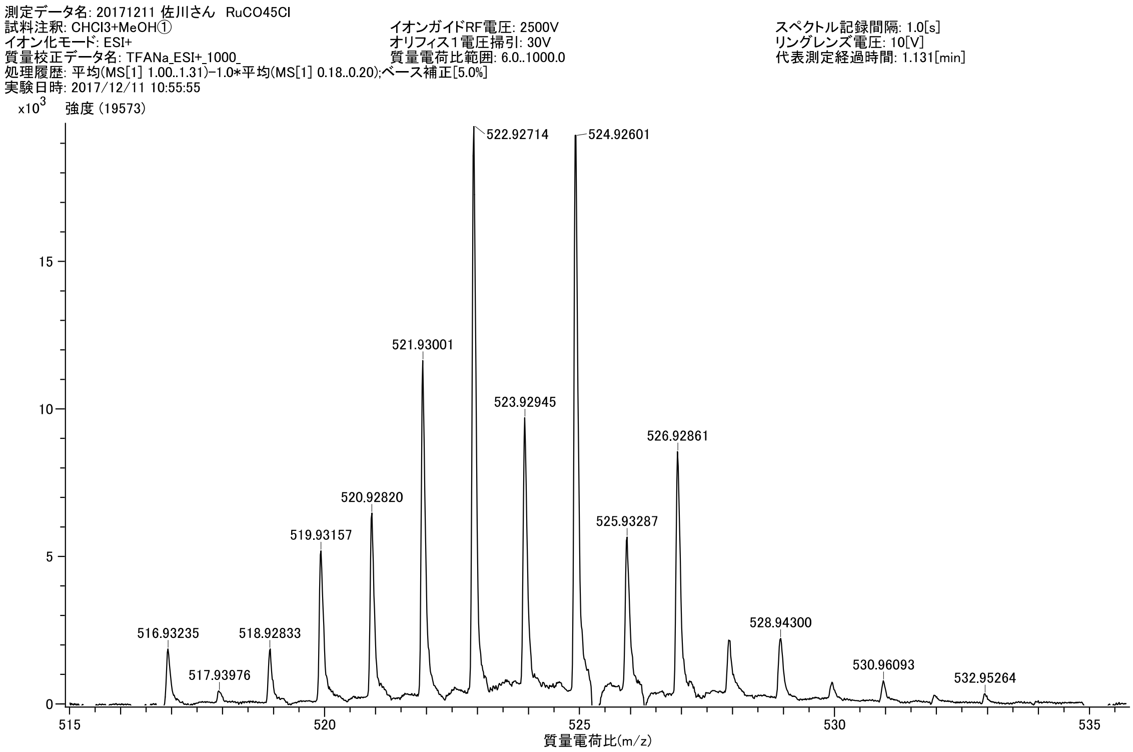

Figure S26. High resolution mass spectrum (ESI-TOF, positive) of **2e**.

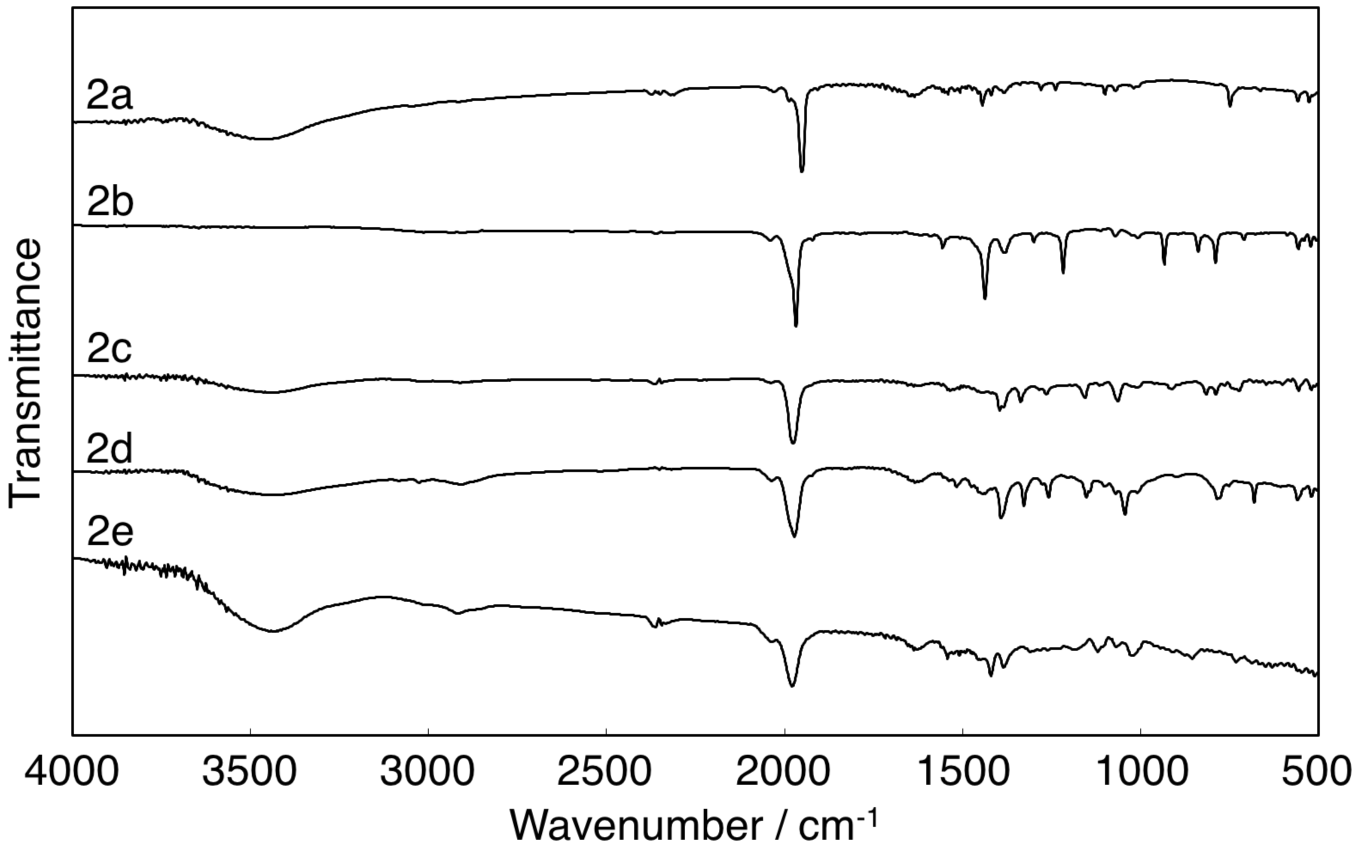

Figure S27. IR spectra of **2a**-**2e** in a KBr pellet.

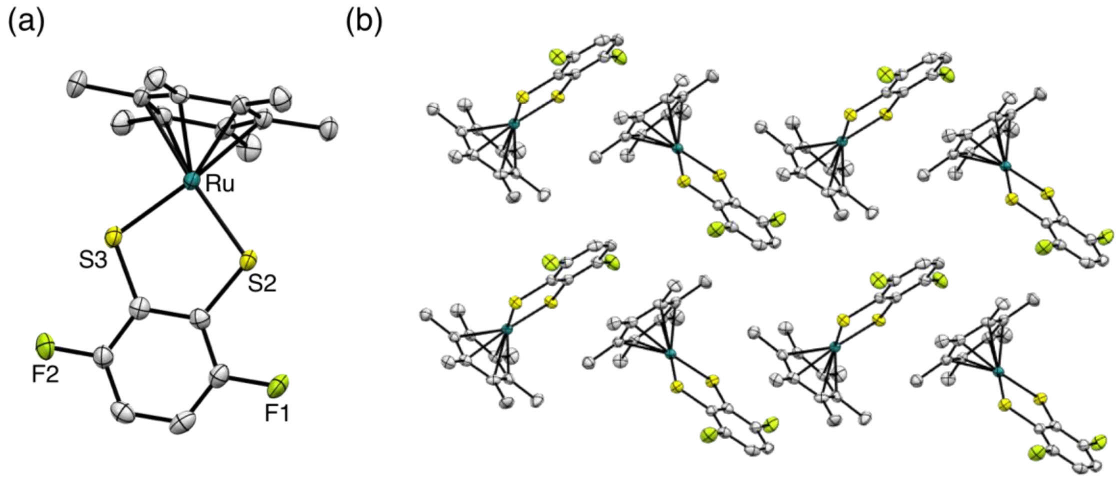

Figure S28. (a) ORTEP drawing of **1b** with thermal ellipsoids at the 50% probability level. Hydrogen atoms are omitted for clarity. Color codes: light grey, C; yellow-green, F; yellow, S; turquoise blue, Ru. (b) Packing structure of **1b**.

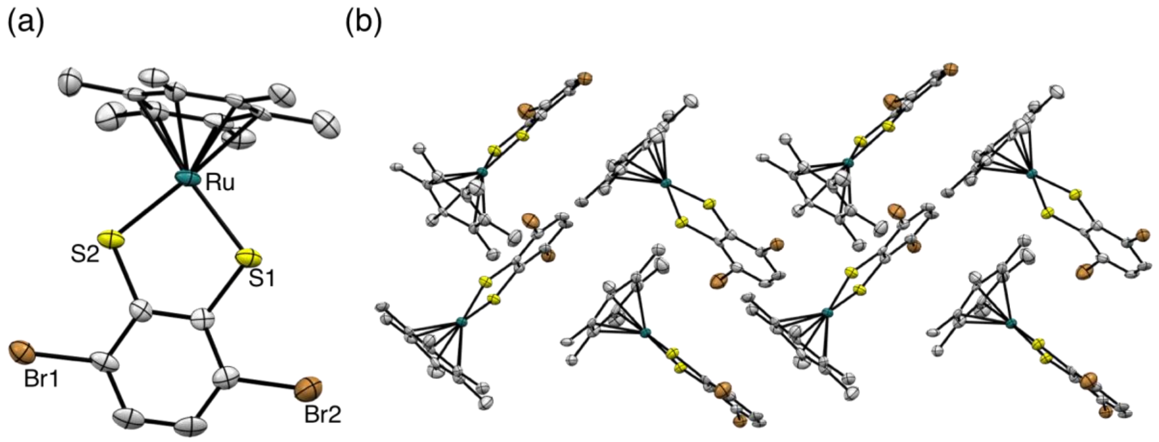

Figure S29. (a) ORTEP drawing of **1d** with thermal ellipsoids at the 50% probability level. Hydrogen atoms are omitted for clarity. Color codes: light grey, C; yellow, S; brown, Br; turquoise blue, Ru. (b) Packing structure of **1d**.

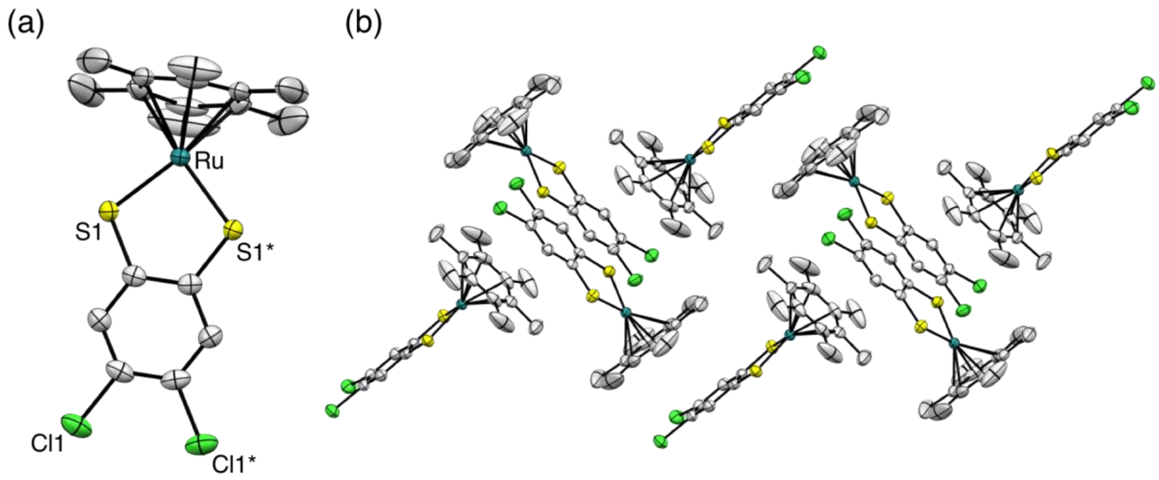

Figure S30. (a) ORTEP drawing of **1e** with thermal ellipsoids at the 50% probability level. Hydrogen atoms are omitted for clarity. Color codes: light grey, C; yellow, S; green, Cl; turquoise blue, Ru. (b) Packing structure of **1e**.

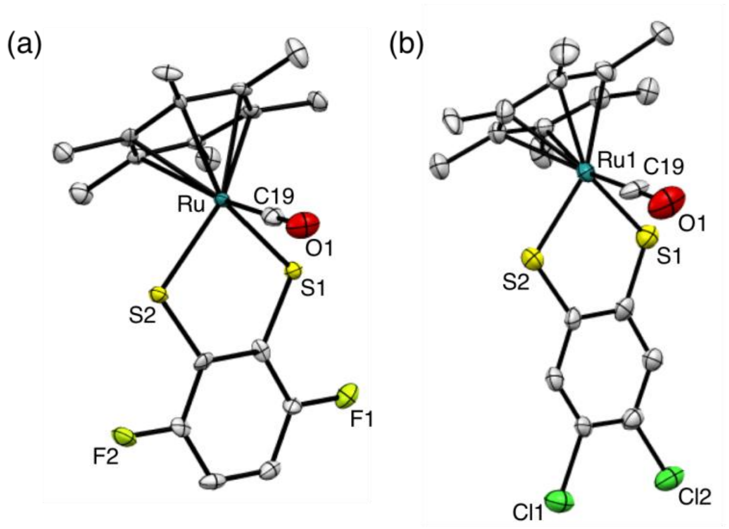

Figure S31. ORTEP drawing of (a) **2b** and (b) **2e** (only one of the three independent molecules is shown) with thermal ellipsoids at the 50% probability level. Hydrogen atoms are omitted for clarity. Color codes: light grey, C; red, O; yellow-green, F; yellow, S; green, Cl; turquoise blue, Ru.

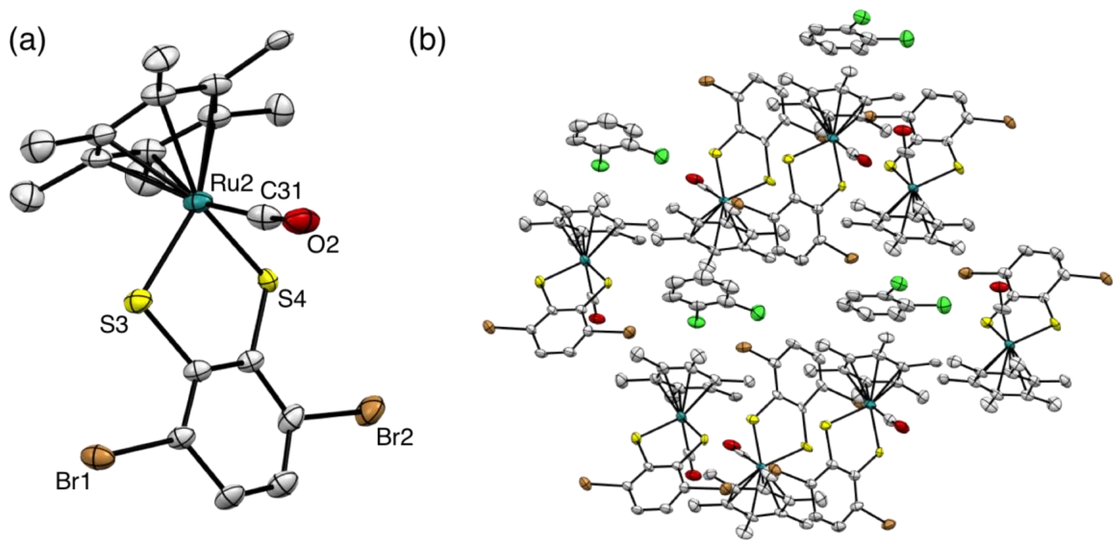

Figure S32. (a) ORTEP drawing of **2d** (only one of the two independent molecules is shown) with thermal ellipsoids at the 50% probability level. Hydrogen atoms and 1,2-dichlorobenzene molecules are omitted for clarity. Color codes: light grey, C; red, O; yellow, S; brown, Br; turquoise blue, Ru. (b) Packing structure of **2d**.

Table S1. Summary of Crystal data of **1b**–**1e**.

	**1b**	**1c**	**1d**	**1e**
Empirical formula	$C_{18}H_{20}F_2RuS_2$	$C_{18}H_{20}Cl_2RuS_2$	$C_{18}H_{20}Br_2RuS_2$	$C_{18}H_{20}Cl_2RuS_2$
Formula weight	439.53	472.43	561.35	472.43
Crystal system	Orthorhombic	Orthorhombic	Orthorhombic	Orthorhombic
Space group	Pca2(1)	Pbca	Pbca	Pnma
a / Å	8.4193(12)	8.3943(9)	8.4049(16)	14.2932(12)
b / Å	14.613(2)	14.2517(14)	14.219(3)	13.6681(13)
c / Å	14.0866(19)	30.219(3)	31.096(6)	8.9038(8)
α / deg	90.00	90.00	90.00	90.00
β / deg	90.00	90.00	90.00	90.00
γ / deg	90.00	90.00	90.00	90.00
V / Å^3	1733.1(4)	3615.2(6)	3716.3(12)	1866.7(3)
Z	4	8	8	4
T / K	103	103	103	103
D_{calcd} / g cm^{-3}	1.685	1.736	2.007	1.681
μ (Mo K_a) / mm^{-1}	1.160	1.390	5.365	1.346
F (000)	888	1904	2192	952
Reflection collected	8183	37682	19567	9242
Independent reflections	2852 (R_{int} = 0.0291)	3645 (R_{int} = 0.1785)	3801 (R_{int} = 0.2257)	1790 (R_{int} = 0.0437)
$R1$ ($I > 2\sigma(I)$)	0.0362	0.0896	0.0978	0.0368
w$R2$ (all data)	0.0937	0.1976	0.2238	0.0953

Table S2. Summary of Crystal data of **2b**–**2e**.

	**2b**	**2c•CH_2Cl_2**	**2d•1,2-$C_6H_4Cl_2$**	**2e**
Empirical formula	$C_{19}H_{20}F_2ORuS_2$	$C_{20}H_{22}Cl_4ORuS$	$C_{22}H_{22}Br_2ClORuS_2$	$C_{19}H_{20}Cl_2ORuS_2$
Formula weight	467.54	585.37	662.86	500.44
Crystal system	Monoclinic	Monoclinic	Monoclinic	Triclinic
Space group	P2(1)/n	P2(1)/n	Cc	P-1
a / Å	11.3499(11)	8.5032(10)	20.583(3)	15.275(4)
b / Å	14.2921(14)	18.179(2)	13.0758(17)	15.561(4)
c / Å	11.6441(11)	14.1179(16)	17.037(2)	15.619(4)
α / deg	90.00	90.00	90.00	119.543(4)
β / deg	109.564(2)	97.789(2)	99.157(2)	93.483(5)
γ / deg	90.00	90.00	90.00	111.135(4)
V / Å^3	1779.8(3)	2162.2(4)	4526.8(10)	2879.6(12)
Z	4	4	8	6
T / K	103	103	103	103
D_{calcd} / g cm^{-3}	1.745	1.798	1.945	1.731
μ (Mo K$_a$) / mm^{-1}	1.140	1.423	4.538	1.318
F (000)	944	1176	2600	1512
Reflection collected	9218	12093	11015	14709
Independent reflections	3259 (R_{int} = 0.0491)	4396 (R_{int} = 0.1158)	5983 (R_{int} = 0.0262)	10120 (R_{int} = 0.0857)
$R1$ ($I > 2\sigma(I)$)	0.0800	0.0878	0.0600	0.1359
w$R2$ (all data)	0.1566	0.2049	0.2260	0.3778
Goodness of fit on F^2	1.340	1.084	1.191	1.094

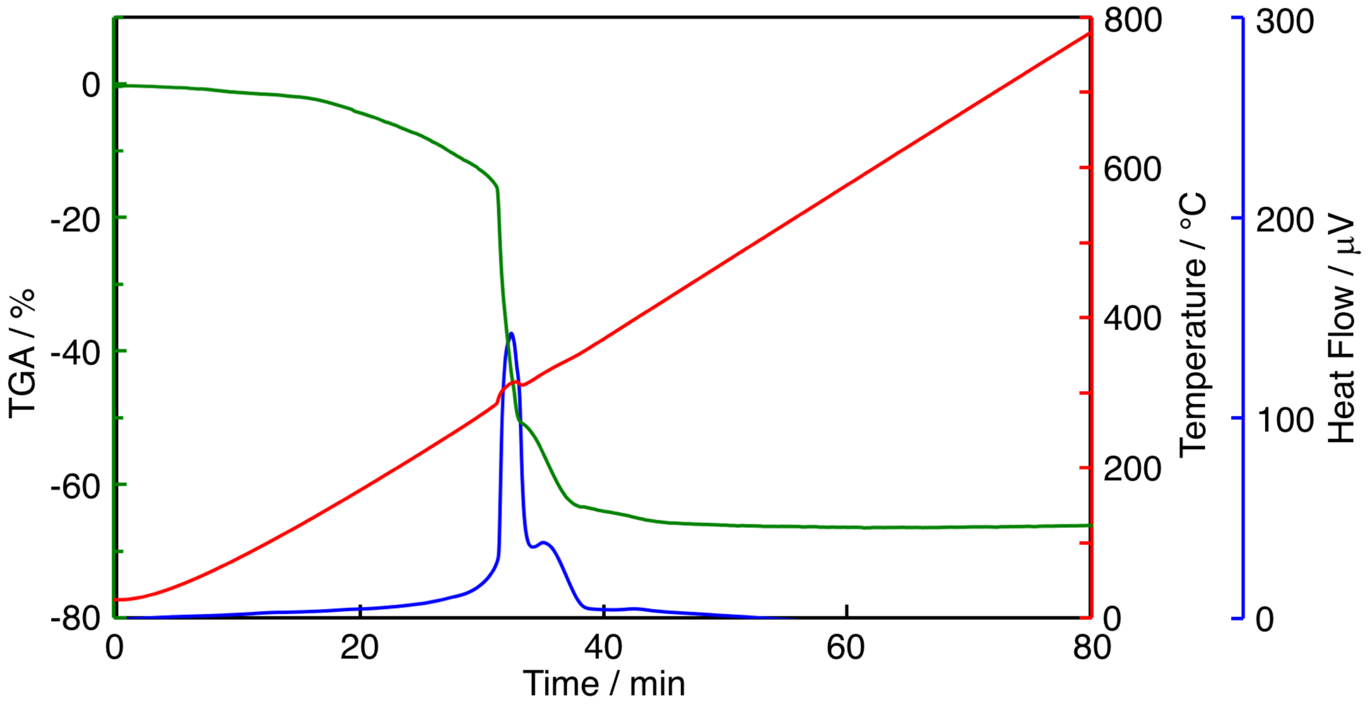

Figure S33. TG-DTA curves of **1c**.

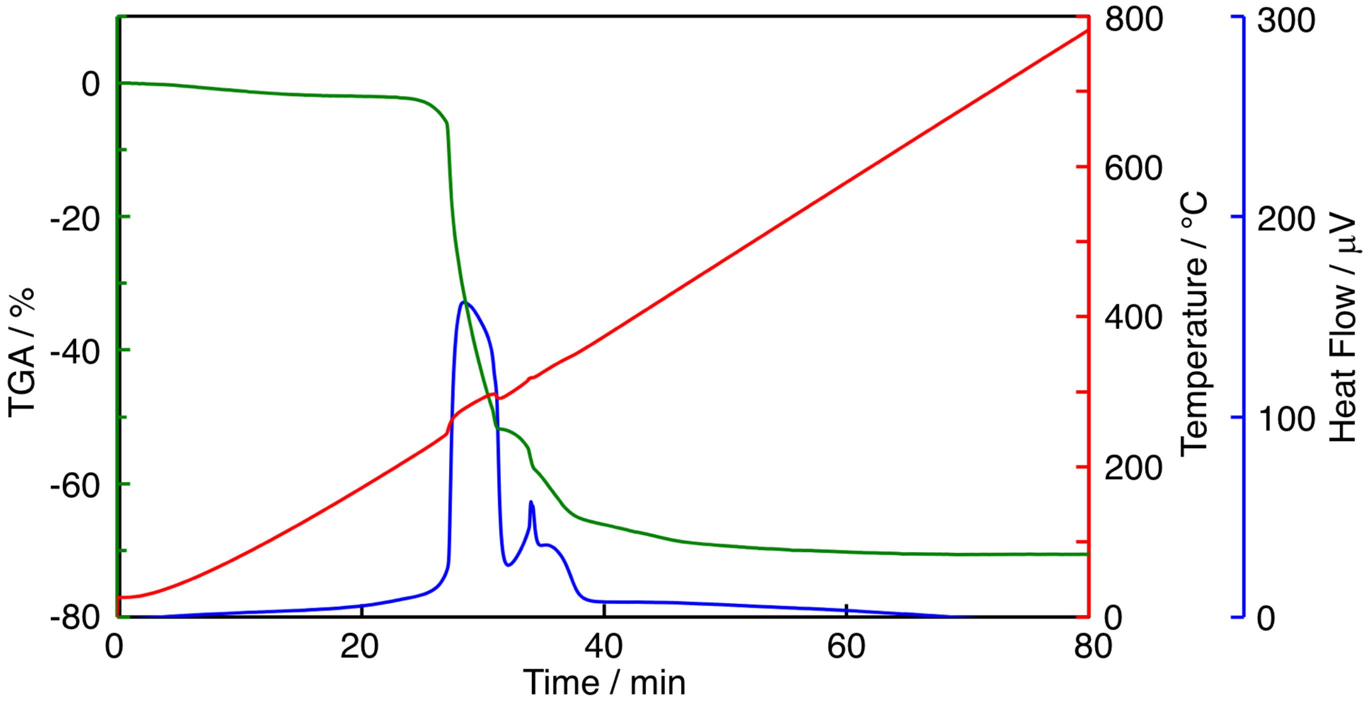

Figure S34. TG-DTA curves of **1e**.

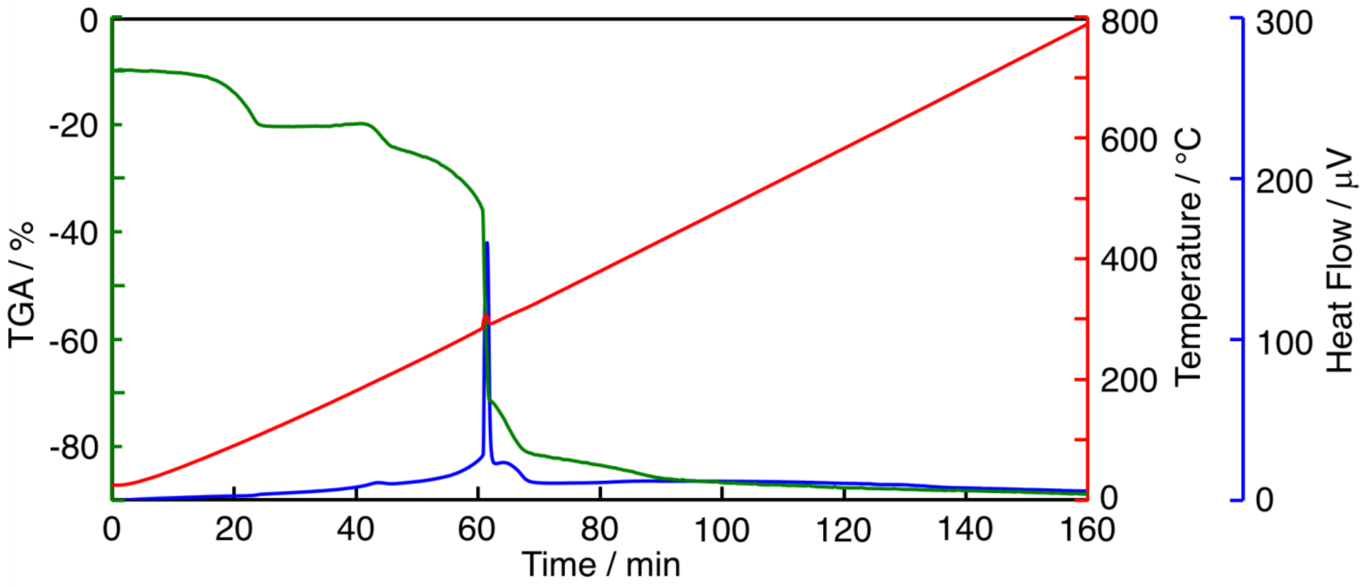

Figure S35. TG-DTA curves of **2c**.

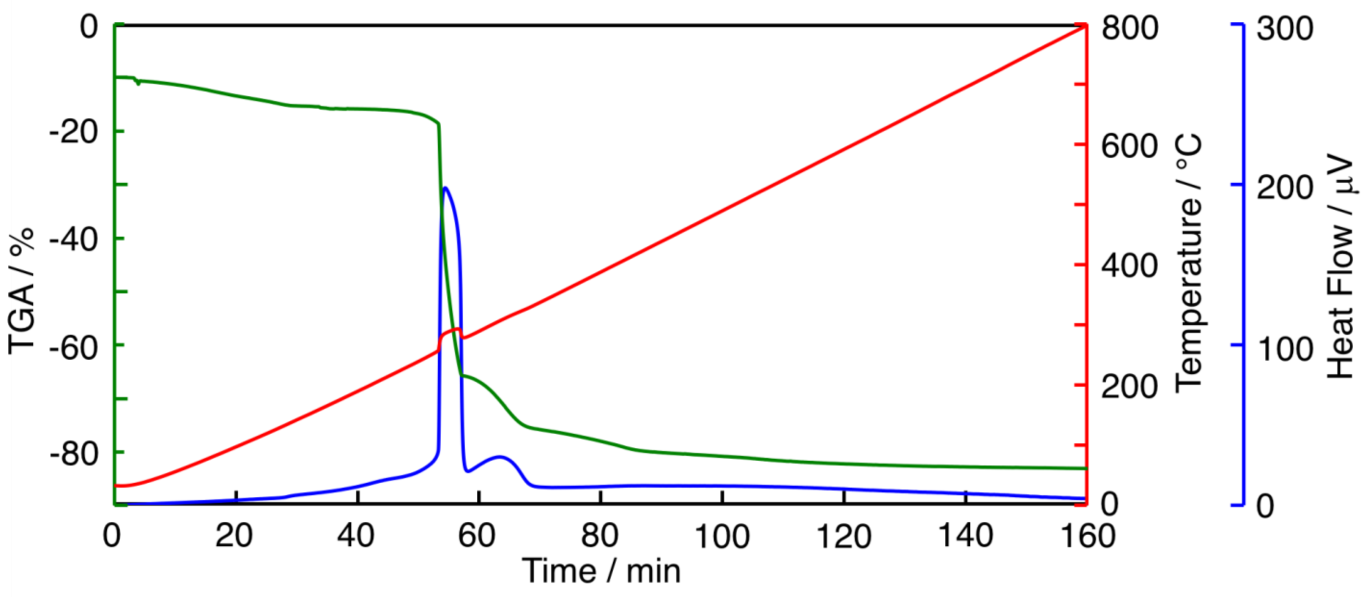

Figure S36. TG-DTA curves of **2e**.

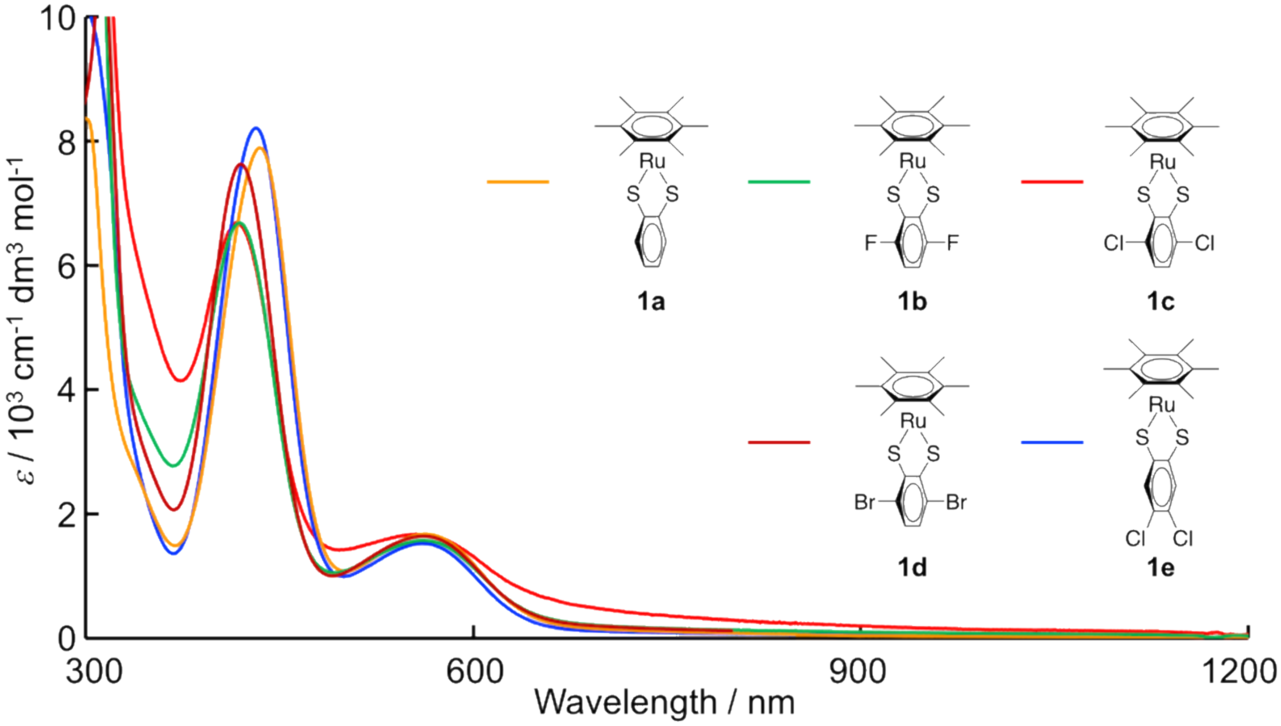

Figure S37. UV-Vis-NIR spectra of **1a-1e** in THF.

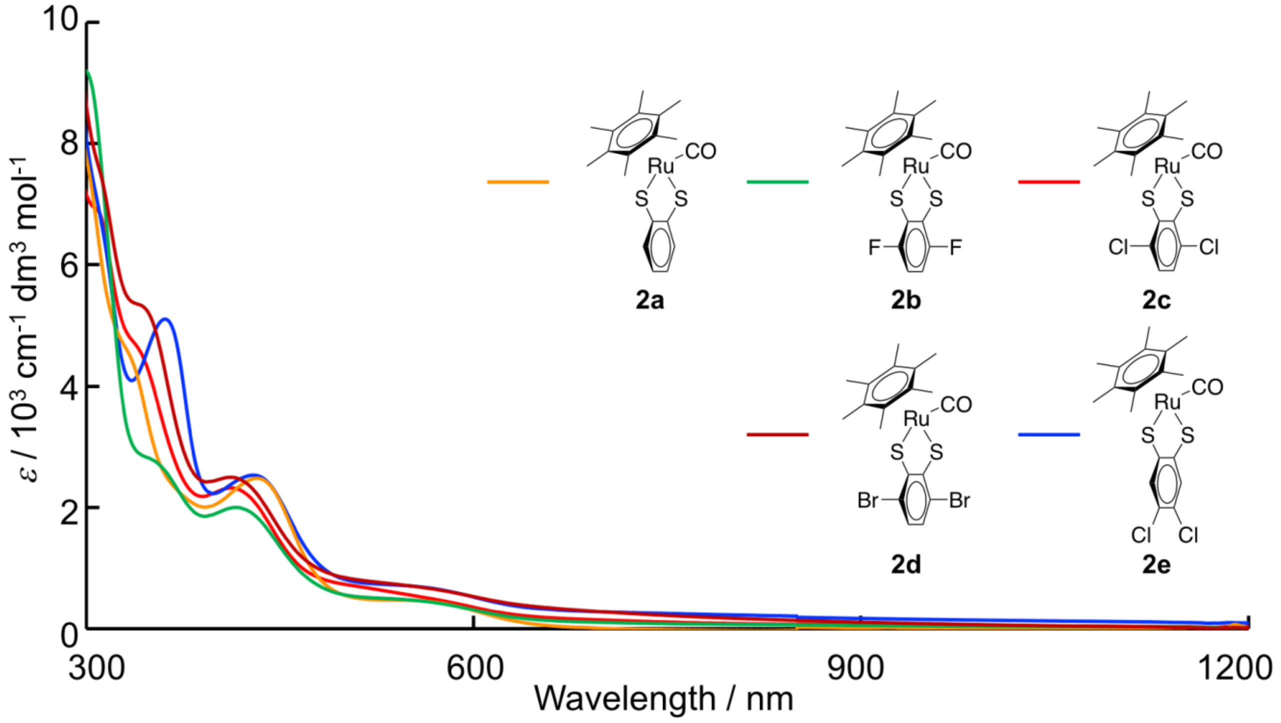

Figure S38. UV-Vis-NIR spectra of **2a-2e** in THF.

Computational Details

The three-parameterized Becke-Lee-Yang-Parr (B3LYP) hybrid exchange-correlation functional was employed for the theoretical calculations. A mixture of basis sets (i.e., Lanl2DZ for Ru, 6-31G(d,p) for H, C, O, S, and halogen atoms) [1–3] was used. Initial structures were taken from the relevant single-crystal X-ray models. Solvent effects were evaluated by means of the conductor-like polarized continuum model (CPCM). The TD-DFT method was used to calculate the excited states related to the absorption spectra. This calculation was implemented using the Gaussian 09W (Revision-A.02) program [4].

Table S3. Cartesian coordinates in optimized geometry of **1c**
(Total energy: – au).

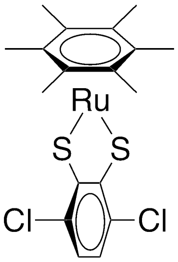

	Coordinates (Angstroms)		
Element	X	Y	Z
Ru	0.966423	0.00707	-0.000199
S	-0.685888	1.593049	0.001975
S	-0.706932	-1.564378	0.002039
C	-3.472665	-1.35307	0.005789
C	-4.663603	0.740829	0.007378
C	2.785285	1.441058	-0.002524
C	2.757129	-1.43397	-0.002407
C	-4.672455	-0.657552	0.007411
C	2.684595	0.725554	1.232783
C	2.681578	0.725507	-1.237556
C	-2.231318	-0.682607	0.00409
C	-3.454797	1.420555	0.005722
C	2.709782	-0.721844	1.235512
C	2.879491	2.953215	-0.00257
C	2.738986	-1.500659	2.535313
C	2.77634	-2.945582	-0.002411
C	2.706488	-0.721915	-1.240254
C	-2.222726	0.733417	0.004055
C	2.58653	1.484682	2.537649
C	2.732153	-1.500874	-2.540024
C	2.580366	1.48456	-2.542233
H	-5.594991	1.295467	0.008639
H	-5.610867	-1.200282	0.008697
H	1.892392	3.430111	-0.001402
H	3.420585	3.30932	-0.88062

H	3.422584	3.309199	0.8743
H	3.561923	-2.222436	2.522467
H	1.810897	-2.056838	2.694032
H	2.890631	-0.854063	3.397699
H	2.276508	-3.356896	-0.879259
H	2.278289	-3.356883	0.875458
H	3.81015	-3.315425	-0.003479
H	3.578096	1.629582	2.987015
H	1.963875	0.954223	3.258232
H	2.133435	2.464706	2.393042
H	3.556055	-2.221623	-2.529897
H	2.879918	-0.854282	-3.40307
H	1.80423	-2.058258	-2.695389
H	2.128167	2.46483	-2.396548
H	1.955489	0.954358	-3.261081
H	3.570787	1.628879	-2.994296
Cl	-3.535185	-3.110773	0.005906
Cl	-3.49391	3.178939	0.005754

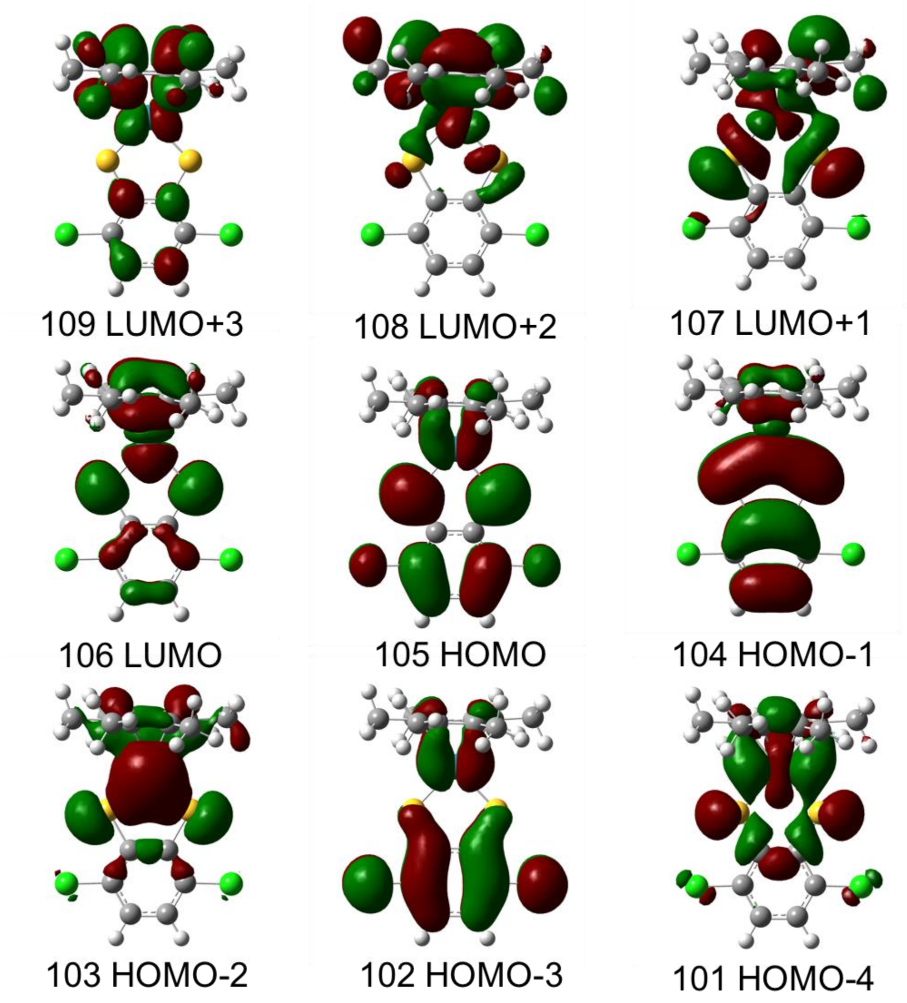

Figure S39. Frontier orbitals (LUMO+3 – HOMO-4) of **1c** estimated by DFT calculation.

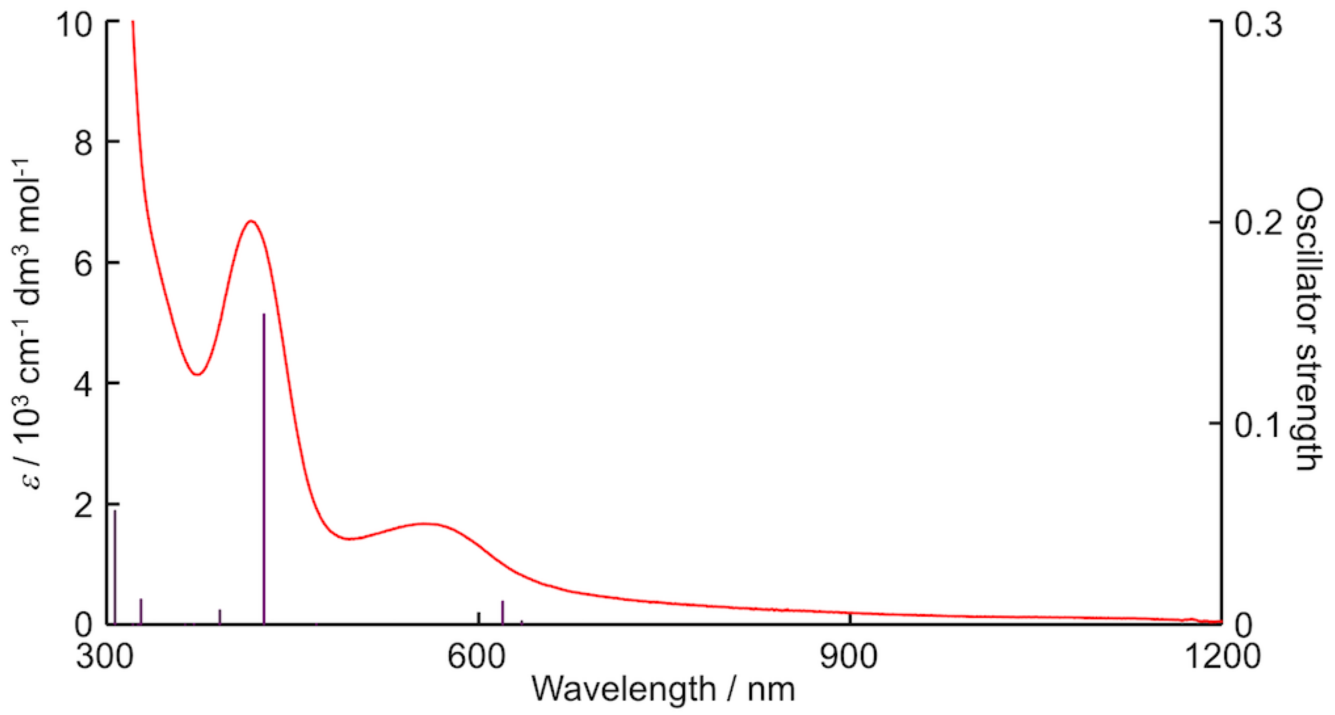

Figure S40. UV-Vis-NIR spectrum of **1c** in THF and calculated oscillator strength (*f*).

Table S5. Calculated absorptions of **1c** at the TD-DFT (B3LYP) level.

Transition	MO	CI Coef.	Transition energy		*f*
			eV	nm	
<1>	103 -> 106	0.69316	1.9514	635.35	0.0016
<2>	105 -> 106	0.64532	2.0007	619.70	0.0112
	102 -> 106	0.22174			
<3>	101 -> 106	0.66385	2.6424	469.20	0.0002
<4>	104 -> 106	0.68573	2.9043	426.90	0.1542
<5>	99 -> 106	0.14677	3.1684	391.31	0.0070
	102 -> 106	0.62893			
<6>	101 -> 106	0.2116	3.3463	370.51	0.0001
	105 -> 107	0.64366			
<7>	100 -> 106	0.69901	3.4110	363.48	0.0000
<8>	103 -> 107	0.65478	3.7823	327.80	0.0124
<9>	104 -> 107	0.68681	3.8113	325.30	0.0000
<10>	105 -> 108	0.66827	3.8538	321.72	0.0000
<11>	101 -> 107	0.65456	3.8640	320.87	0.0000
<12>	105 -> 109	0.61655	4.0444	306.56	0.0563

Table S6. Cartesian coordinates in optimized geometry of **2c** (Total energy: – au).

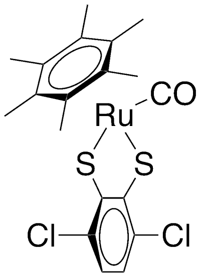

Element	Coordinates (Angstroms)		
	X	Y	Z
Ru	0.910652	-0.090128	-0.243708
S	-0.873845	-1.610183	0.017081
S	-0.730479	1.600557	-0.386859
O	0.862314	-0.45052	-3.21379
C	3.223774	0.54497	-0.049988
C	2.479943	1.400758	0.805016
C	1.831635	0.863488	1.98927
C	1.76125	-0.523044	2.172005
C	2.338163	-1.40883	1.177363
C	3.131748	-0.883111	0.124569
C	1.016306	-1.16111	3.317459
C	2.218909	-2.901093	1.389238
C	3.901728	-1.814835	-0.789082
C	4.14099	1.100552	-1.121454
C	2.465983	2.899362	0.588645
C	1.177455	1.842617	2.931955
C	0.876407	-0.313082	-2.063522
C	-2.351056	-0.650844	-0.200623
C	-3.61187	-1.272288	-0.191536
C	-4.790436	-0.550093	-0.348539
C	-4.728655	0.830532	-0.522484
C	-3.489588	1.462809	-0.536198
C	-2.288183	0.750339	-0.3773
H	1.603251	-1.963335	3.773015
H	0.08223	-1.602212	2.944922
H	0.757196	-0.45384	4.101865
H	2.87088	-3.224207	2.211084
H	2.501773	-3.468775	0.505044
H	1.196057	-3.181999	1.647296
H	4.734552	-2.280304	-0.24834
H	4.316047	-1.295944	-1.651143
H	3.267753	-2.616908	-1.170886

H	5.155215	0.713811	-0.976793
H	4.204029	2.185843	-1.085417
H	3.822503	0.819645	-2.129371
H	3.270326	3.389113	1.152874
H	1.519486	3.33138	0.913011
H	2.58338	3.157116	-0.463006
H	0.910923	1.394072	3.886403
H	0.257372	2.234	2.477633
H	1.835144	2.691289	3.137273
H	-5.744497	-1.064477	-0.335899
H	-5.633634	1.413813	-0.648129
Cl	-3.735202	-3.013886	0.038277
Cl	-3.457418	3.210941	-0.745986

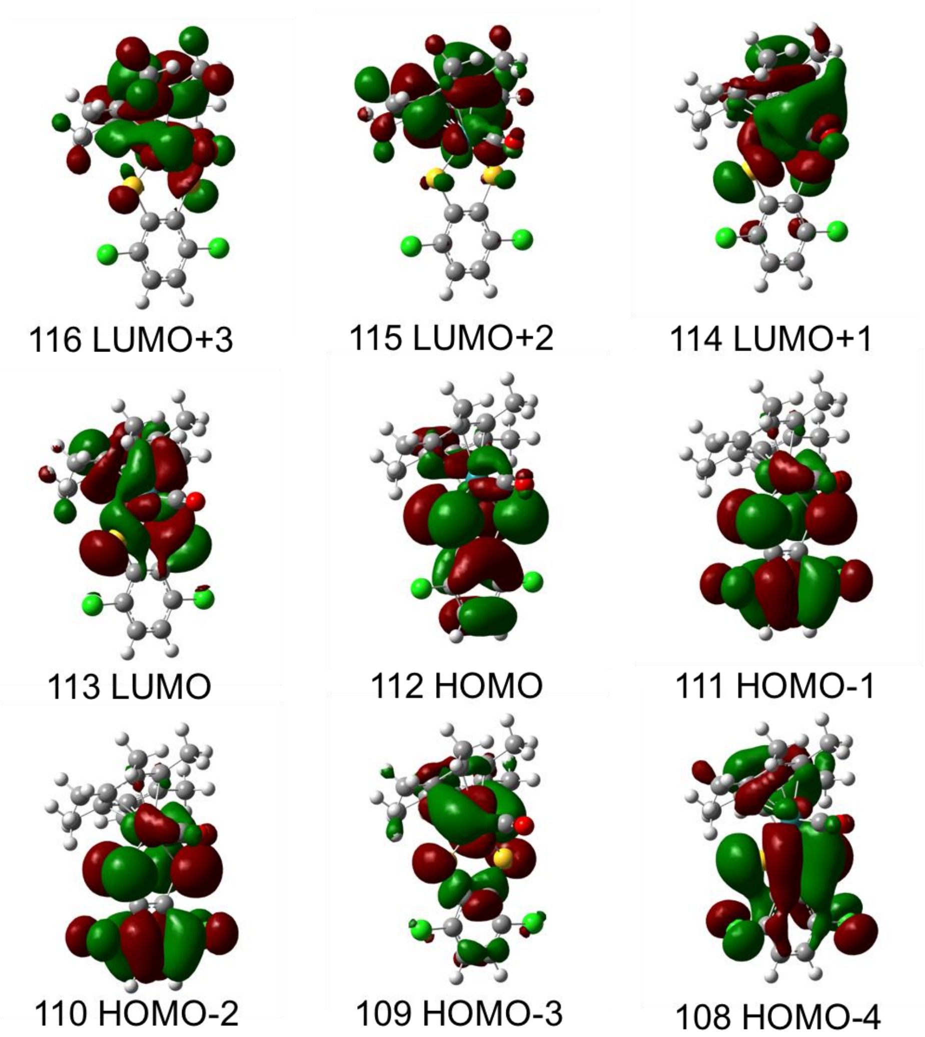

Figure S41. Frontier orbitals (LUMO+3 – HOMO-4) of **2c** estimated by DFT calculation.

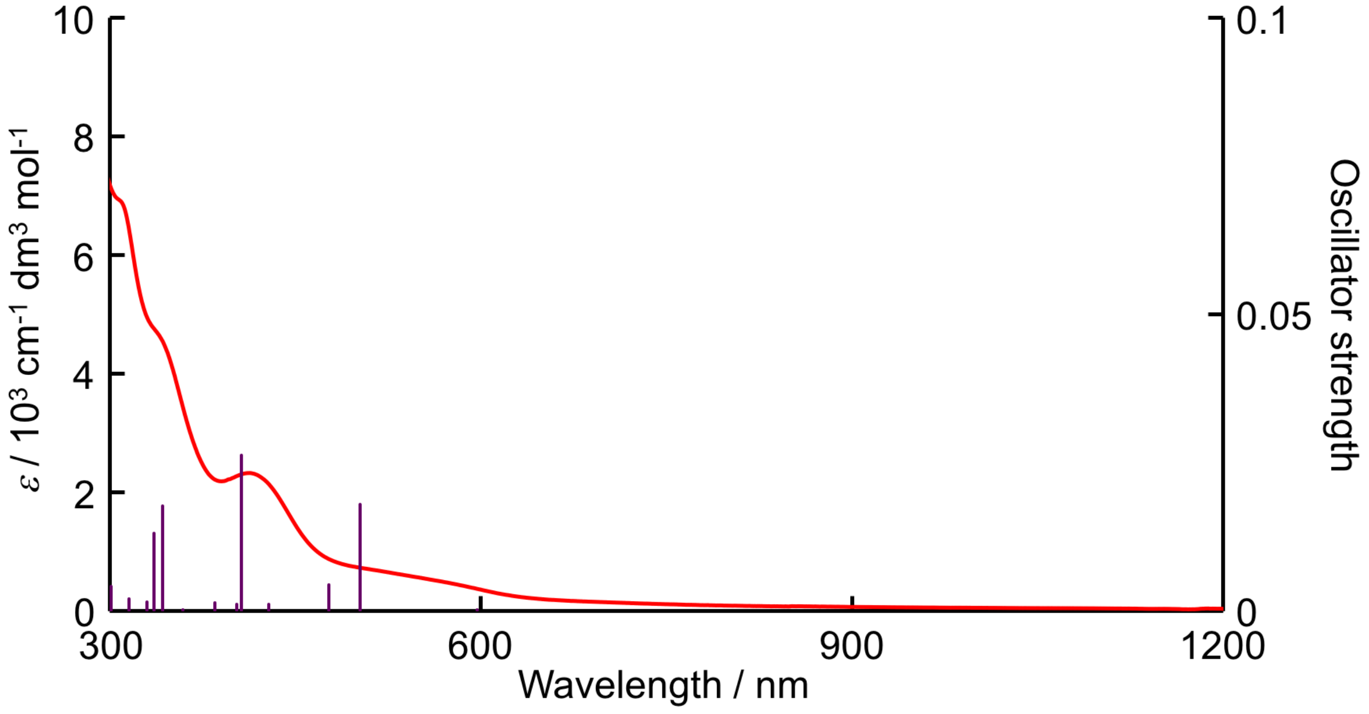

Figure S42. UV-Vis-NIR spectrum of **2c** in THF and calculated oscillator strength (*f*).

Table S7. Calculated absorptions of **2c** at the TD-DFT (B3LYP) level.

Transition	MO	CI Coef.	Transition energy		*f*
			eV	nm	
<1>	112 -> 113	0.69314	2.0772	596.89	0.0001
<2>	112 -> 114	0.57843	2.4674	502.49	0.0179
<3>	111 -> 113	0.57381	2.5996	476.94	0.0044
	112 -> 114	0.37263			
<4>	111 -> 114	0.63807	2.8936	428.48	0.0011
<5>	112 -> 115	0.69078	3.0513	406.33	0.0262
<6>	109 -> 113	0.64656	3.0791	402.66	0.0011
<7>	112 -> 116	0.69275	3.2222	384.78	0.0013
<8>	111 -> 115	0.68336	3.4553	358.83	0.0001
<9>	109 -> 114	0.32851	3.6206	342.44	0.0176
	110 -> 113	0.53120			
<10>	109 -> 114	0.48763	3.6959	335.46	0.0131
	111 -> 116	0.45077			
<11>	110 -> 113	0.37098	3.7611	329.65	0.0015
	111 -> 116	0.47691			
<12>	110 -> 114	0.59867	3.9319	315.33	0.0020
	111 -> 114	0.23604			
<13>	112 -> 117	0.66682	4.1166	301.18	0.0041
<14>	107 -> 113	0.49331	4.1247	300.59	0.0006

Table S8. Cartesian coordinates in optimized geometry of **1e** (Total energy: – au).

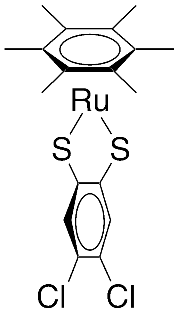

Element	Coordinates (Angstroms)		
	X	Y	Z
Ru	-1.283474	0.001942	0.000053
S	0.386829	1.583641	-0.000382
S	0.383984	-1.590486	0.000213
C	3.1304	-1.401314	0.000162
C	4.336258	0.698467	-0.000252
C	-3.08623	1.449087	-0.001296
C	-3.079538	-1.425302	0.00168
C	4.334393	-0.711177	0.000036
C	-2.992481	0.73156	-1.236483
C	-2.992341	0.734173	1.235376
C	1.908262	-0.70784	0.000009
C	3.133828	1.391351	-0.000396
C	-3.02885	-0.715094	-1.237639
C	-3.169082	2.962067	-0.003018
C	-3.063969	-1.495424	-2.536334
C	-3.108693	-2.936765	0.002399
C	-3.028237	-0.712572	1.239598
C	1.910463	0.70015	-0.000257
C	-2.888403	1.488526	-2.542108
C	-3.062686	-1.490137	2.539985
C	-2.888043	1.494153	2.539248
H	3.136999	-2.485813	0.000394
H	3.142905	2.475807	-0.000601
H	-2.178727	3.431855	-0.004028
H	-3.708318	3.323117	0.87414
H	-3.709082	3.320983	-0.880596
H	-3.890284	-2.213267	-2.521272
H	-2.1388	-2.056121	-2.696178
H	-3.214064	-0.849032	-3.399173
H	-2.619196	-3.350929	0.883644
H	-2.606274	-3.351543	-0.871399
H	-4.144924	-3.299673	-0.005133
H	-3.878908	1.64138	-2.991157
H	-2.270628	0.952046	-3.26244

H	-2.427078	2.464873	-2.398762
H	-3.892114	-2.204505	2.52879
H	-3.207468	-0.84152	3.402017
H	-2.139385	-2.054318	2.698172
H	-2.427162	2.470334	2.393411
H	-2.269692	0.959696	3.260575
H	-3.878409	1.647652	2.98837
Cl	5.833578	1.606502	-0.000412
Cl	5.829372	-1.622997	0.000254

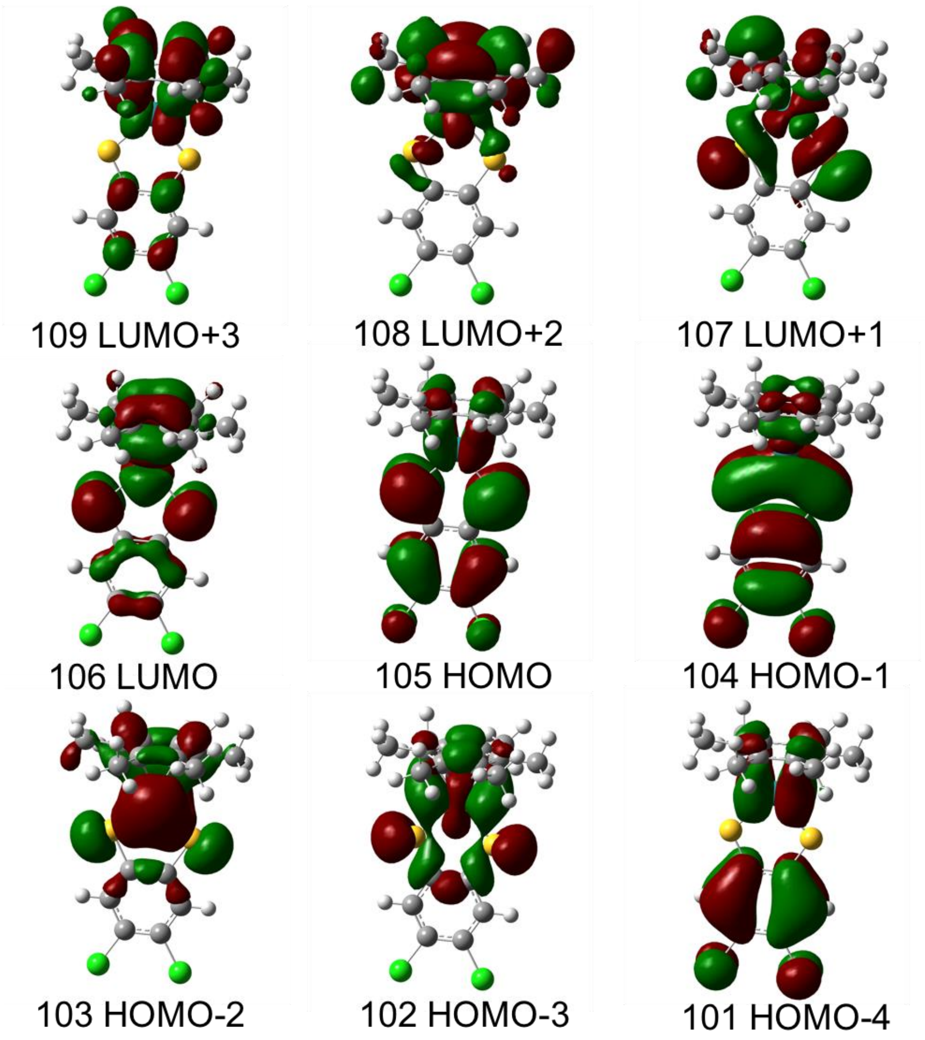

Figure S43. Frontier orbitals (LUMO+3 – HOMO-4) of **1e** estimated by DFT calculation.

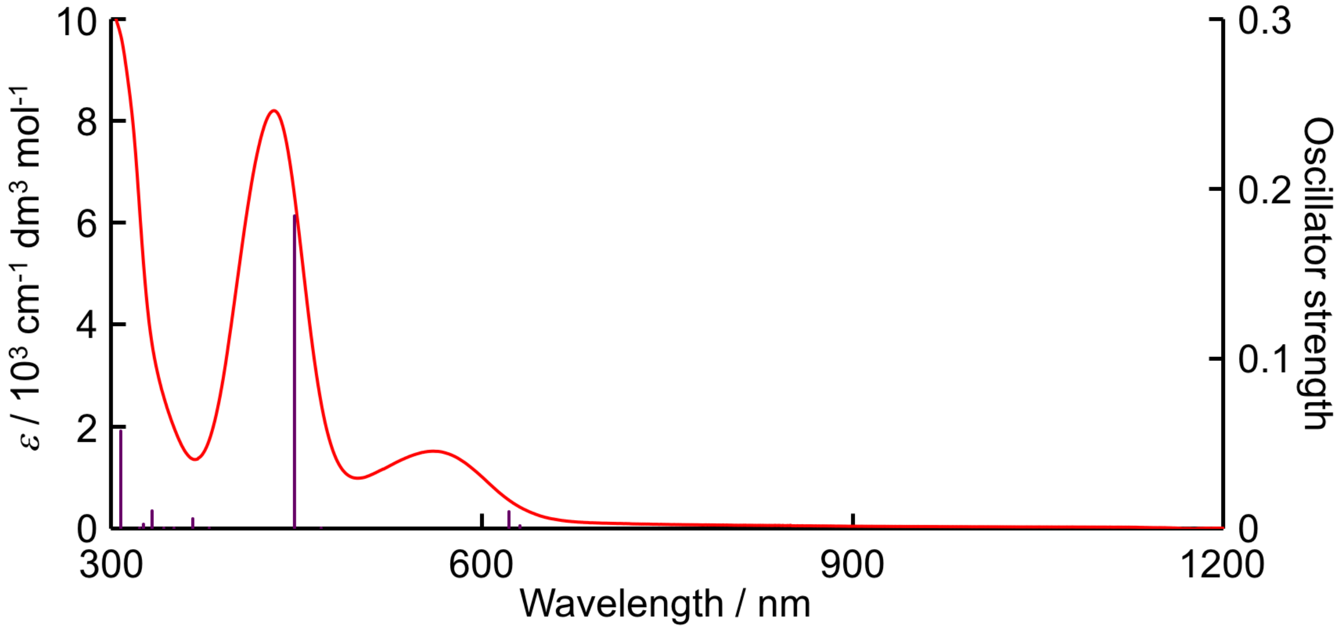

Figure S44. UV-Vis-NIR spectrum of **1e** in THF and calculated oscillator strength (*f*).

Table S9. Calculated absorptions of **1e** at the TD-DFT (B3LYP) level.

Transition	MO	CI Coef.	Transition energy		*f*
			eV	nm	
<1>	103 -> 106	0.69227	1.9667	630.40	0.0017
<2>	101 -> 106	0.21152	1.9944	621.65	0.0098
	105 -> 106	0.65398			
<3>	102 -> 106	0.65332	2.6378	470.02	0.0003
<4>	104 -> 106	0.69093	2.7649	448.42	0.1840
<5>	102 -> 106	0.23735	3.2675	379.45	0.0001
	105 -> 107	0.64649			
<6>	101 -> 106	0.61812	3.3866	366.11	0.0055
<7>	100 -> 106	0.63959	3.5318	351.06	0.0000
	104 -> 107	0.28577			
<8>	104 -> 107	0.63069	3.6223	342.28	0.0001
<9>	103 -> 107	0.64319	3.7257	332.78	0.0105
<10>	102 -> 107	0.65671	3.8007	326.22	0.0023
<11>	105 -> 109	0.67969	3.8362	323.19	0.0000
<12>	105 -> 109	0.61957	4.0310	307.58	0.0572

Table S10. Cartesian coordinates in optimized geometry of **2e** (Total energy: – au).

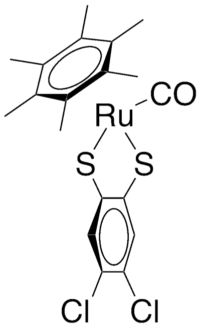

Element	Coordinates (Angstroms)		
	X	Y	Z
Ru	-1.25442	-0.00336	-0.402545
S	0.44311	1.623516	-0.121716
S	0.446442	-1.628448	-0.115989
O	-0.837125	-0.019055	-3.365691
C	-3.547408	-0.714169	-0.418719
C	-2.893284	-1.419144	0.626595
C	-2.42646	-0.707326	1.805907
C	-2.420636	0.691769	1.812119
C	-2.882598	1.416972	0.641118
C	-3.521467	0.727275	-0.422577
C	-1.852841	1.503386	2.948893
C	-2.834943	2.927995	0.666083
C	-4.196734	1.501266	-1.536278
C	-4.30043	-1.443032	-1.513832
C	-2.807678	-2.930857	0.611055
C	-1.87445	-1.528566	2.944017
C	-0.994368	-0.010732	-2.218016
C	1.960827	0.70232	-0.086327
C	3.18013	1.391637	-0.052669
C	4.388888	0.703202	0.003829
C	4.390142	-0.69975	0.006319
C	3.182677	-1.39058	-0.047892
C	1.961966	-0.703766	-0.084116
H	-2.514263	2.333453	3.211531
H	-0.88716	1.929302	2.645371
H	-1.68595	0.91532	3.848349
H	-3.611589	3.324402	1.333038
H	-2.995909	3.363025	-0.318365
H	-1.870413	3.286122	1.030217
H	-5.097745	2.002165	-1.162386
H	-4.49426	0.858685	-2.362463
H	-3.539446	2.269752	-1.946845

H	-5.338703	-1.095946	-1.542038
H	-4.326519	-2.517855	-1.348991
H	-3.868378	-1.26907	-2.503375
H	-3.671137	-3.382078	1.117061
H	-1.906315	-3.276254	1.11663
H	-2.766484	-3.322224	-0.404553
H	-1.750831	-0.95187	3.857982
H	-0.890353	-1.928884	2.665381
H	-2.527151	-2.375184	3.172361
H	3.190144	2.476169	-0.068975
H	3.194654	-2.47514	-0.0603
Cl	5.879378	1.622016	0.057274
Cl	5.882297	-1.61567	0.063201

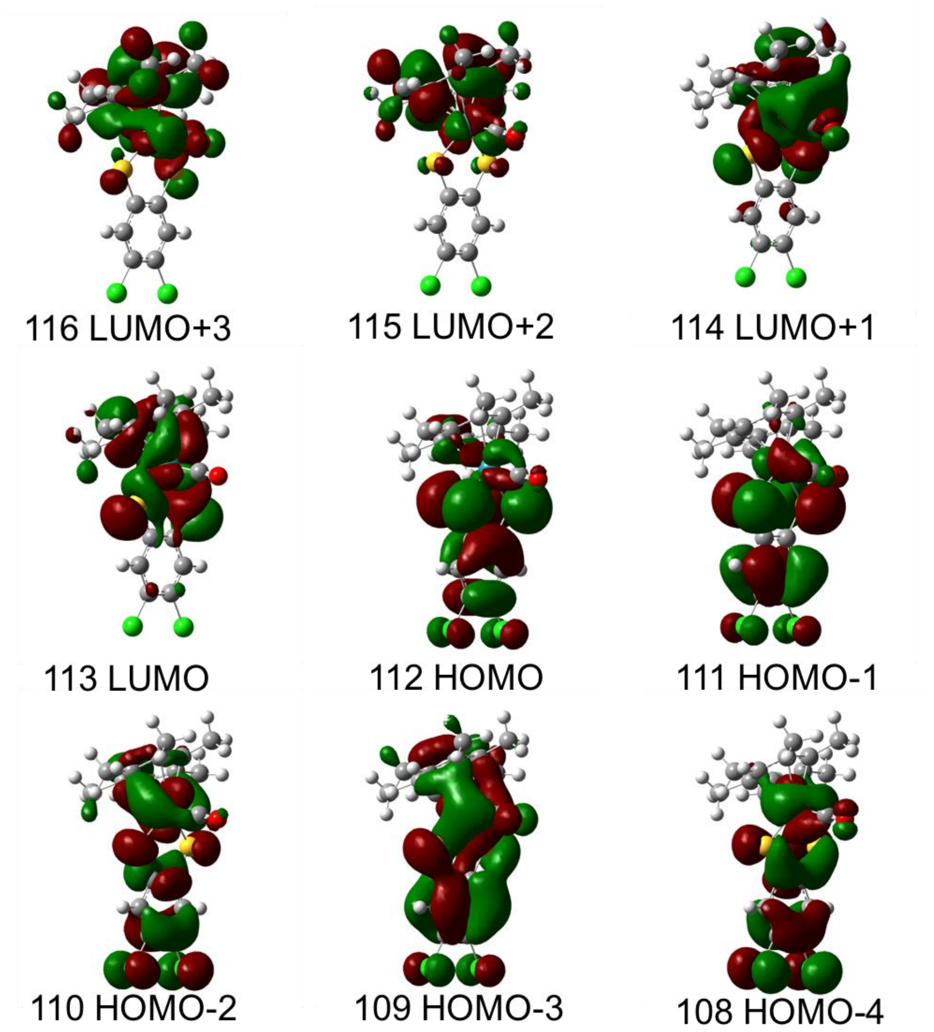

Figure S45. Frontier orbitals (LUMO+3 – HOMO-4) of **2e** estimated by DFT calculation.

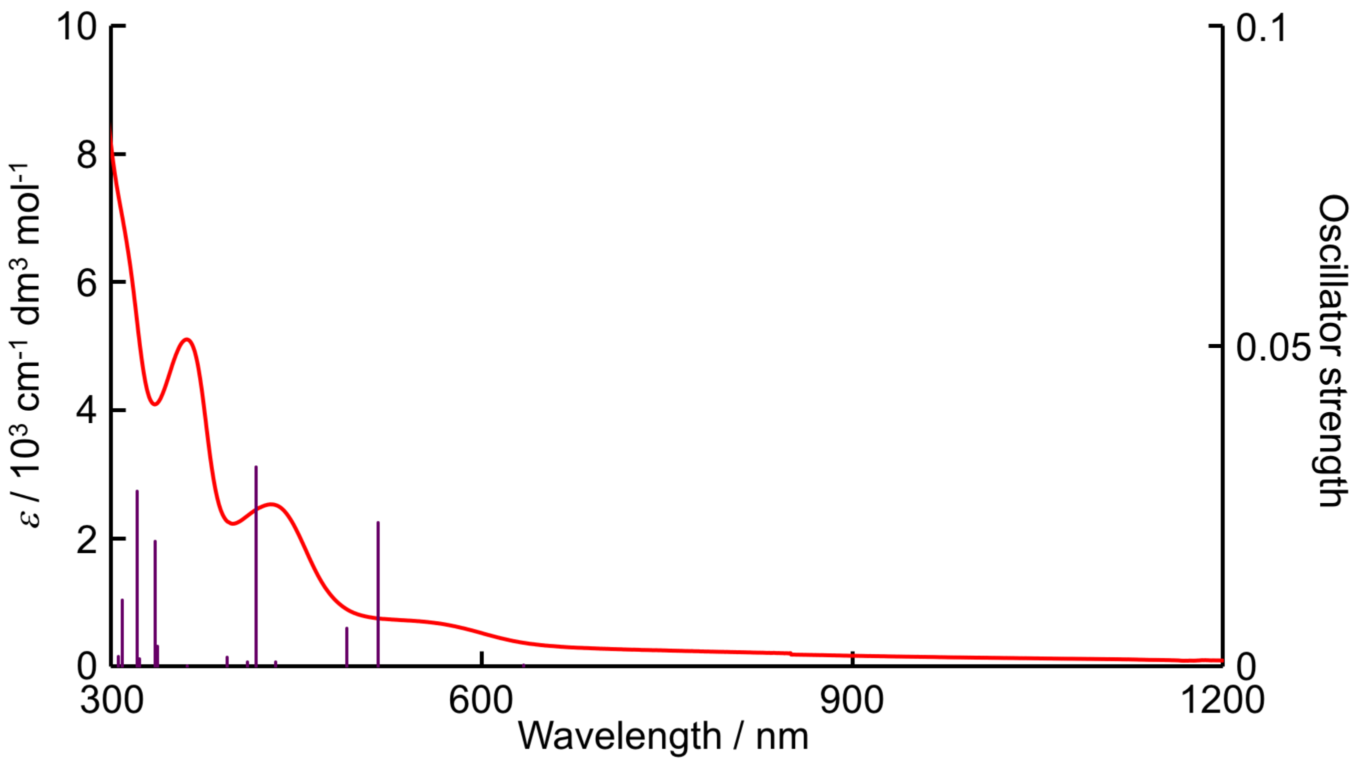

Figure S46. UV-Vis-NIR spectrum of **2e** in THF and calculated oscillator strength (*f*).

Table S11. Calculated absorptions of **2e** at the TD-DFT (B3LYP) level.

Transition	MO	CI Coef.	Transition energy		*f*
			eV	nm	
<1>	112 -> 113	0.69358	1.9549	634.21	0.0002
<2>	112 -> 114	0.54590	2.4027	516.03	0.0225
<3>	111 -> 113	0.54923	2.5246	491.11	0.0060
	112 -> 114	0.41708			
<4>	111 -> 114	0.65002	2.8603	433.46	0.0007
<5>	112 -> 115	0.69298	2.9718	417.20	0.0312
<6>	108 -> 113	0.40062	3.0203	410.50	0.0007
	110 -> 116	0.52464			
<7>	112 -> 116	0.68996	3.1490	393.72	0.0014
<8>	111 -> 115	0.68900	3.4259	361.91	0.0001
<9>	108 -> 114	0.35466	3.6747	337.40	0.0031
	110 -> 114	0.54691			
<10>	111 -> 116	0.65241	3.6930	335.73	0.0195
<11>	108 -> 113	0.48792	3.8398	322.89	0.0012
<12>	109 -> 113	0.59746	3.8620	321.04	0.0273
<13>	112 -> 117	0.52901	4.0149	308.81	0.0104
	112 -> 118	0.37173			
<14>	112 -> 119	0.63507	4.0573	305.58	0.0016
<15>	109 -> 114	0.57816	4.1194	300.98	0.0011
	112 -> 119	0.20809			

References

1 Becke, A.D. Density-functional exchange-energy approximation with correct asymptotic behavior. *Phys. Rev. A* **1988**, *38*, 3098–3100.

2 Becke, A.D. Density-functional thermochemistry. III. The role of exact exchange. *J. Chem. Phys.* **1993**, *98*, 5648–5652.

3 Perdew, J.P.; Wang, Y. Accurate and simple analytic representation of the electron-gas correlation energy. *Phys. Rev. B* **1992**, *45*, 13244–13249.

4 Frisch, M.J.; Trucks, G.W.; Schlegel, H.B.; Scuseria, G.E.; Robb, M.A.; Cheeseman, J.R.; Scalmani, G.; Barone, V.; Mennucci, B.; Petersson, G.A.; Nakatsuji, H.; Caricato, M.; Li, X.; Hratchian, H.P.; Izmaylov, A.F.; Bloino, J.; Zheng, G.; Sonnenberg, J.L.; Hada, M.; Ehara, M.; Toyota, K.; Fukuda, R.; Hasegawa, J.; Ishida, M.; Nakajima, T.; Honda, Y.; Kitao, O.; Nakai, H.; Vreven, T.; Montgomery, J.A., Jr.; Peralta, J.E.; Ogliaro, F.; Bearpark, M.; Heyd, J.J.; Brothers, E.; Kudin, K.N.; Staroverov, V.N.; Kobayashi, R.; Normand, J.; Raghavachari, K.; Rendell, A.; Burant, J.C.; Iyengar, S.S.; Tomasi, J.; Cossi, M.; Rega, N.; Millam, J.M.; Klene, M.; Knox, J.E.; Cross, J.B.; Bakken, V.; Adamo, C.; Jaramillo, J.; Gomperts, R.; Stratmann, R.E.; Yazyev, O.; Austin, A.J.; Cammi, R.; Pomelli, C.; Ochterski, J.W.; Martin, R.L.; Morokuma, K.; Zakrzewski, V.G.; Voth, G.A.; Salvador, P.; Dannenberg, J.J.; Dapprich, S.; Daniels, A.D.; Farkas, O.; Foresman, J.B.; Ortiz, J.V.; Cioslowski, J.; Fox, D.J. *Gaussian 09*, Revision A.02; Gaussian, Inc.: Wallingford, CT, USA, 2009.
